# Adaptive sample size determination for the development of clinical prediction models

**DOI:** 10.1186/s41512-021-00096-5

**Published:** 2021-03-22

**Authors:** Evangelia Christodoulou, Maarten van Smeden, Michael Edlinger, Dirk Timmerman, Maria Wanitschek, Ewout W. Steyerberg, Ben Van Calster

**Affiliations:** 1 Department of Development & Regeneration, KU Leuven, Leuven, Belgium; 2 Julius Center for Health Sciences and Primary Care, University Medical Center Utrecht, Utrecht, Netherlands; 3 Department of Medical Statistics, Informatics, and Health Economics, Medical University Innsbruck, Innsbruck, Austria; 4 Department of Obstetrics and Gynecology, University Hospitals Leuven, Leuven, Belgium; 5 University Clinic of Internal Medicine III - Cardiology and Angiology, Tirol Kliniken, Innsbruck, Austria; 6 Department of Biomedical Data Sciences, Leiden University Medical Center, Leiden, Netherlands; 7 EPI-centre, KU Leuven, Leuven, Belgium

**Keywords:** Adaptive design, Clinical prediction models, Events per variable, Model development, Model validation, Sample size

## Abstract

**Background:**

We suggest an adaptive sample size calculation method for developing clinical prediction models, in which model performance is monitored sequentially as new data comes in.

**Methods:**

We illustrate the approach using data for the diagnosis of ovarian cancer (*n* = 5914, 33% event fraction) and obstructive coronary artery disease (CAD; *n* = 4888, 44% event fraction). We used logistic regression to develop a prediction model consisting only of a priori selected predictors and assumed linear relations for continuous predictors. We mimicked prospective patient recruitment by developing the model on 100 randomly selected patients, and we used bootstrapping to internally validate the model. We sequentially added 50 random new patients until we reached a sample size of 3000 and re-estimated model performance at each step. We examined the required sample size for satisfying the following stopping rule: obtaining a calibration slope ≥ 0.9 and optimism in the c-statistic (or AUC) < = 0.02 at two consecutive sample sizes. This procedure was repeated 500 times. We also investigated the impact of alternative modeling strategies: modeling nonlinear relations for continuous predictors and correcting for bias on the model estimates (Firth’s correction).

**Results:**

Better discrimination was achieved in the ovarian cancer data (c-statistic 0.9 with 7 predictors) than in the CAD data (c-statistic 0.7 with 11 predictors). Adequate calibration and limited optimism in discrimination was achieved after a median of 450 patients (interquartile range 450–500) for the ovarian cancer data (22 events per parameter (EPP), 20–24) and 850 patients (750–900) for the CAD data (33 EPP, 30–35). A stricter criterion, requiring AUC optimism < = 0.01, was met with a median of 500 (23 EPP) and 1500 (59 EPP) patients, respectively. These sample sizes were much higher than the well-known 10 EPP rule of thumb and slightly higher than a recently published fixed sample size calculation method by Riley et al. Higher sample sizes were required when nonlinear relationships were modeled, and lower sample sizes when Firth’s correction was used.

**Conclusions:**

Adaptive sample size determination can be a useful supplement to fixed a priori sample size calculations, because it allows to tailor the sample size to the specific prediction modeling context in a dynamic fashion.

**Supplementary Information:**

The online version contains supplementary material available at 10.1186/s41512-021-00096-5.

## Background

Clinical prediction models, such as diagnostic and prognostic models, are ubiquitous in the literature [1–3]. A prerequisite for developing a robust and useful prediction model is to have a sufficient sample size that allows for adequate model complexity but avoids overfitting the development data. An overfit model captures random noise in the data to generate risk estimates, because the noise was misinterpreted as predictive signal. A well-known rule of thumb is to have a minimum of 10 events per variable (EPV) in the smallest outcome group [4], although EPV > 20 has also been suggested [5]. Strictly, these rules refer to events per considered model coefficient (excluding intercept) in a regression analysis, although it is sometimes incorrectly interpreted in terms of events per variable in the final model (i.e., excluding variables eliminated by any data-driven variable selection procedure). We will use the term “events per candidate predictor parameter” (EPP) instead, in line with a recent publication [6].

The 10 EPP rule of thumb has shortcomings [4, 6–13]. Most importantly, the rule does not guarantee decent risk model performance [9]. For example, the rule does not reflect the impact of the event fraction of the outcome (prevalence or incidence) and the underlying predictive strength on the required sample size [14]. Recently, a comprehensive method for a priori fixed sample size calculations for prediction model development was proposed, integrating the number of candidate parameters, the assumed event fraction and the anticipated Cox-Snell R-squared [6]. This is an important advance, because it requires more detailed argumentation of the anticipated modeling context and it focuses specifically on prediction model performance.

We aimed to extend a priori fixed sample size calculations with an adaptive approach that dynamically learns from model performance as new data comes in. As such, sample size can be tailored gradually to all specifics of the prediction modeling context at hand. This adaptive method requires that a model development strategy is prespecified (e.g., which predictors to consider, how to select predictors, how to address nonlinearity, how to deal with possible interactions between predictors) before data are collected and that the data can be accessed and analyzed while data collection is ongoing.

We apply the approach to two case studies. The case studies involve the diagnosis of ovarian cancer and the diagnosis of obstructive coronary artery disease (CAD). We empirically study the stability of the proposed adaptive method and illustrate it for different model development strategies.

## Methods

### Case studies

The first case study involves the development of a prediction model to diagnose malignancy in women presenting with an ovarian tumor who were selected for surgical removal of the tumor. Such a model can support decisions about type and location of surgery, e.g., whether referral to a specialized gynecological oncology unit is warranted. We use a dataset including 5914 women recruited between 1999 and 2012 into cohort studies from the International Ovarian Tumor Analysis (IOTA) consortium [15]. In total, 1931 women had a malignant mass (33% prevalence). We developed a model using 7 a priori selected predictors: age (years), maximum lesion diameter (mm), maximum diameter of the largest solid component (mm), number of papillations (0, 1, 2, 3, > 3), presence of acoustic shadows (binary), presence of ascites (binary), and presence of bilateral masses (binary) (see Table 1).

**Table 1:** Descriptive characteristics of the variables from the ovarian cancer and coronary artery disease datasets

Predictor	Statistics	Result	Missing values (%)
*Ovarian cancer case study*			
Age (years)	Mean (range)	48 (8-96)	0
Maximum lesion diameter (mm)	Mean (range)	82 (8-760)	0
Maximum diameter of solid part (mm)	Mean (range)	28 (0-380)	0
Number of papillations (0-4)	Mean (range)	0.4 (0-4)	0
Acoustic shadows (no/yes)	*N* yes (%)	743 (13%)	0
Ascites (no/yes)	*N* yes (%)	720 (12%)	0
Bilateral masses (no/yes)	*N* yes (%)	1141 (19%)	0
*Coronary artery disease case study*			
Age (years)	Mean (range)	64 (18-89)	0
HDL cholesterol (mg/dL)	Mean (range)	56 (15-188)	312 (6.4%)
LDL cholesterol (mg/dL)	Mean (range)	128 (21-341)	310 (6.3%)
Logarithm of fibrinogen (log(mg/dL))	Mean (range)	5.9 (4.6-7.3)	119 (4.1%)
Sex (male/female)	*N* male (%)	3028 (62%)	0
Chest pain (no/yes)	*N* yes (%)	2987 (61%)	0
Diabetes mellitus (no/yes)	*N* yes (%	757 (16%)	0
Hypertension (no/yes)	*N* yes (%)	3730 (76%)	0
Dyslipidaemia (no/yes)	*N* yes (%)	3115 (64%)	0
c-Reactive protein > 1.00 mg/dL (no/yes)	*N* yes (%)	681 (14%)	96 (2%)
Smoking status			640 (13%)
Never	*N* (%)	2304 (54%)	
Former	*N* (%)	1088 (26%)	
Current	*N* (%)	856 (20%)	

The second case study deals with the development of a prediction model to diagnose obstructive CAD in symptomatic patients. Such a model can support selection of patients for coronary angiography. We use the Coronary Artery disease Risk Determination In Innsbruck by diaGnostic ANgiography (CARDIIGAN) dataset, a cohort study consisting of 4888 patients with suspected CAD, of which 2127 (44%) had obstructive CAD [16]. A model with 11 a priori selected predictors was developed: sex (binary), age (years), diabetes mellitus (binary), HDL cholesterol (mg/dL), LDL cholesterol (mg/dL), logarithm of fibrinogen (mg/dL), c-reactive protein > 1.00 mg/dL (binary), hypertension (binary), dyslipidaemia (binary), chest pain (binary), and ever smoking status (categorical) (see Table 1). Some predictors suffered from missing data. We used single stochastic imputation (based on fully conditional specification) for illustrative purposes. We stress that multiple imputation is preferable over single stochastic imputation in many applications. We therefore also address the issue of combining the adaptive sample size procedure with multiple imputation of missing values.

### Adaptive procedure for sample size determination

Upon commencement of a model development study, the modeling strategy needs to be prespecified. The adaptive procedure is as follows:

i. Determine an initial estimation of the required sample size (*N*_0_). This is best done using the recently suggested fixed sample size determination procedure from Riley and colleagues, based on the number of candidate parameters, the assumed outcome event fraction, and the anticipated Cox-Snell R-squared [6].
ii. Determine a sample size *N*_start_ (< *N*_0_) at which performance is estimated for the first time, and recruit *N*_start_ patients in the study. This is the first model development dataset.
iii. Apply the prespecified modeling strategy on the development dataset to obtain model M_D_, evaluate and store apparent performance measures (PM_D_; i.e., performance on exactly the same data that were used to obtain M_D_).
iv. Perform internal validation; we use Harrell’s enhanced bootstrap, a recommended method that has been shown to perform well [17–19]. The enhanced bootstrap works as follows:
  - Draw a bootstrap sample with replacement from the development dataset.
  - Apply the complete modeling strategy on the bootstrap sample resulting in a bootstrap model (M_B_).
  - Store the performance measures of the model when evaluated on the bootstrap dataset (PM_B_).
  - Evaluate the bootstrap model M_B_ on the development dataset and store the performance measures (PM_O_).
  - Calculate the optimism as the difference PM_B_ − PM_O_ for each performance measure.
  - Repeat a–e *B* − 1 times, and calculate the average of the *B* optimism estimates.
  - Subtract the average optimism from the apparent performance PM_D_ to obtain internally validated performance estimates for model M_D_—denoted as “bootstrap-corrected” performance estimates in this study.
v. Recruit *N*_add_ new patients, add them to the development dataset, and repeat steps iii and iv.
vi. Repeat step v until a prespecified stopping rule has been reached (see below); the model M_D_ for which the stopping rule has been reached is the final prediction model.

Prediction modeling in the presence of missing data forms an extra challenge. Prevailing methods such as multiple imputation in combination with bootstrapping adds a layer of computational complexity. We suggest an extension of the adaptive sample size procedure in combination with multiple imputation for missing values in the predictors in Additional file 1: Appendix A, following the recommendation that multiple imputation should be embedded in the bootstrapping procedure [20, 21].

### Resampling study

To evaluate the adaptive sample size procedure, we conducted a resampling study using the two case studies. We sampled without replacement from the available datasets, with *N*_start_ = 100, *B* = 200, and *N*_add_ = 50. We continued until a sample size of 3000 was reached, even when the stopping rule was reached earlier. Learning curves were constructed, which are visual displays of model performance by increasing sample size. In a real-life application, there will only be one learning curve on which to base the sample size. To empirically assess stability of the procedure, we repeated this process 500 times. In the end, an average learning curve was calculated. Continuing to a sample size of 3000 allowed us to show learning curves of fixed length.

For the CAD dataset, we also implemented the adaptive procedure including multiple imputation of missing data. For computation time reasons, we illustrate the result using a single learning curve repetition only.

### Modeling strategies

The basic prediction modeling strategy in our resampling study involved a standard maximum likelihood logistic regression on a prespecified set of predictors. Continuous variables were assumed to have a linear relation with the (logit of the) outcome. This implied 7 parameters for the ovarian cancer study and 12 parameters for the CAD study.

Three alternative prediction modeling strategies were investigated. The first alternative strategy differs from the basic strategy by modeling possibly nonlinear relations of continuous predictor variables with the outcome using restricted cubic splines with three knots (i.e., one additional parameter per predictor) [17]. This implies 3 additional parameters for the ovarian cancer study (age, maximum diameter of lesion, maximum diameter of largest solid component) and 4 additional parameters for the CAD study (age, HDL and LDL cholesterol, and logarithm of fibrinogen). The second strategy differs from the basic strategy because logistic regression with Firth’s correction was used, but without addressing the functional form for continuous predictors [22]. In short, Firth’s correction is targeted at the reduction of the first-order bias in maximum likelihood estimates of coefficients when we fit logistic regression models on small sample sizes. In that manner, problems related to separation are bypassed, and coefficients are shrunk. With the use of this approach, we aimed to investigate how bias-reduction methods affect the model performance at the initial low sample sizes of our methodology when compared to our basic strategy. No intercept correction was applied, because it has no impact on the adopted performance measures in this study (see below). Note that such correction is required in real-life applications. The third strategy differed from the basic strategy by performing backward variable elimination with the default alpha 5%, so requiring statistical significance of predictors as *p* < 0.05. This strategy may generally be considered degenerate, but is still occasionally found in the medical literature, most likely due to its easy implementation [23]. We implemented it as follows. For the ovarian cancer data, we forced the variable age in the model, so that it could not be eliminated. For the CAD data, age and gender were forced in the model. Age (and gender for CAD) are basic predictors of clinical importance for these prediction problems. In addition, including these key predictors avoids the computational burden of a resulting “empty” model (i.e. with no selected predictors).

We fitted these models in R version 4.0.0, using packages stats, rms, brglm, and ModelGood. In Additional file 1: Appendix B, we describe the occurrence and handling of warning and error messages

### Performance measures at internal validation

The key performance criteria for prediction models relate to discrimination (the extent to which the risk estimates are higher for patients with the event than for patients without) and calibration (the accuracy of the risk estimates). We assessed discrimination using the area under the ROC curve (AUC) or c-statistic. Optimism in the AUC was calculated as the apparent AUC minus the bootstrap-corrected AUC as estimated using Harrell’s enhanced bootstrap method. At internal validation, calibration can be investigated with the slope of the linear predictor, which may serve as a shrinkage factor for future predictions [24–27]. A slope below 1 suggests that the estimated risks are too extreme (i.e., too close to 0 and 1). Conversely, a slope above 1 suggests that the estimated risks are too modest (too close to the event fraction). We only used the calibration slope in our study, because the calibration intercept is less relevant at internal validation [28].

### Stopping rules

Formal sample size calculations depend on performance criteria that are specified a priori. For prediction model development, the aim is to avoid suboptimal and optimistic model performance. Therefore, we consider it sensible to base stopping rules on AUC optimism and the calibration slope, both of which are assessed on internal validation. With ever increasing sample size for model development, model optimism will disappear (calibration slope will approach 1, AUC optimism will approach 0). Riley and colleagues reasonably suggested to aim for a shrinkage factor ≥ 0.9; hence, we targeted a calibration slope of ≥ 0.9 [6, 27]. Regarding AUC optimism, a value of at most 0.02 may be a reasonable target. Therefore, a stopping rule could be to reach a calibration slope of at least 0.9 and a AUC optimism of at most 0.02. To reduce the impact of random variation caused by reassessing performance after every 50 new patients, we added the requirement that the calibration slope and AUC optimism targets should be reached on two consecutive performance assessments (stopping rule 1). Regarding AUC optimism, a more strict maximum of 0.01 has been used before [29]. Therefore, we assessed the performance of another stopping rule that requires calibration slope ≥ 0.9 and AUC optimism ≤ 0.01 on two consecutive performance assessments (stopping rule 2).

For each of the 500 repetitions, we determined the sample size at which a stopping rule is satisfied and summarized the final sample size, bootstrap-corrected AUC, and bootstrap-based calibration slope of the final prediction model through their median and interquartile range across the 500 repetitions.

### Holdout performance

As an additional evaluation, we assessed performance of the developed model at each sample size on the patients that were never used for model development in that specific repetition. Each repetition added patients until a sample size of 3000 was reached. This implied that 2914 (ovarian cancer) and 1888 (CAD) patients were not sampled at any stage of a given repetition. These patients served as a holdout sample. We evaluated the AUC and calibration slope of the developed model at each stage on the holdout sample. We compared the average learning curve of internally validated performance with the average performance on the holdout data. Note that this validation step is not possible when applying the methodology in practice.

## Results

### 10 EPP rule and Riley’s methods for initial sample size estimates

For the ovarian cancer data, with 7 predictive parameters and 33% (1931/5914) outcome prevalence, 10 EPP will be reached after on average 215 patients (10*7*(5914/1931)). For the CAD data, with 12 predictive parameters and 44% outcome prevalence, 10 EPP will be reached after on average 276 patients (10*12*(4888/2127)). When using Riley’s method [6], the minimally required sample size was 314 (15 EPP) for the ovarian cancer data and 669 (25 EPP) for the CAD data (Additional file 1: Appendix C). Hence, Riley’s method advises a 46% higher sample size for the ovarian cancer data and a 142% higher sample size for the CAD data than the EPP 10 method. Note that we add 50 patients at a time, such that stopping when 10 EPP is reached will lead to slightly higher observed EPP. For Riley’s method, recruitment is stopped after 350 patients for the ovarian cancer data and 700 for the CAD data.

### Mimicking practice: single learning curve

**Fig. 1:**
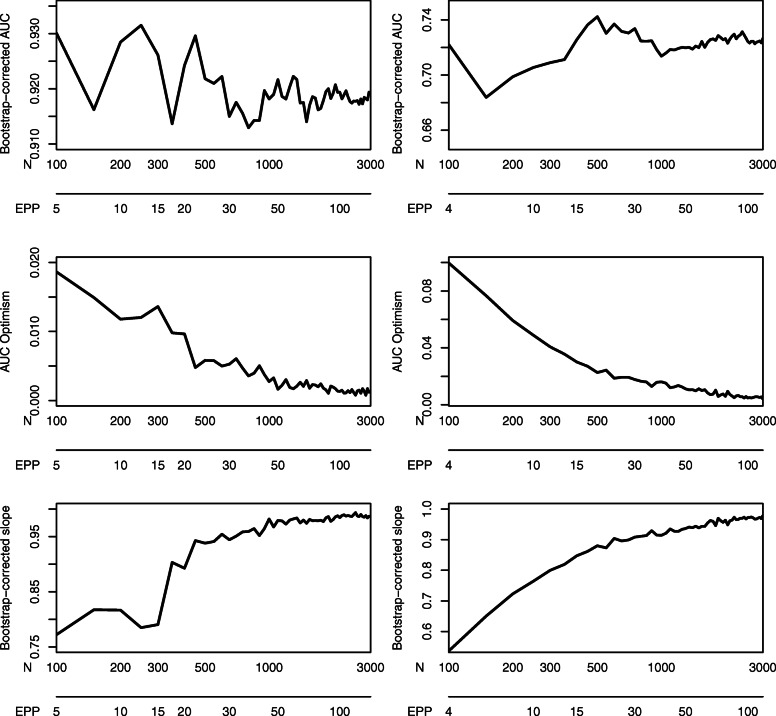
Single learning curves of bootstrap-corrected AUC, AUC optimism, and bootstrap-corrected calibration slope for the ovarian cancer data (left) and the coronary artery disease data (right)

In practice, we can only draw one learning curve to base the required sample size for a study on. Figure 1 shows single learning curves of bootstrap-corrected AUC, AUC optimism, and calibration slope for both case studies. We show the curve for the first of the 500 random repetitions. For both case studies, these plots suggest that a large sample size is required before performance is plateauing. Better prediction was achieved in the ovarian cancer data (bootstrap-corrected AUC at *N* = 3000 slightly above 0.9) than in the CAD data (AUC slightly above 0.7).

Figure S1 (Additional file 1: Appendix D) shows learning curves for the CAD case study where we used multiple imputation to address missing values rather than a single imputation.

### Assessing stability for the basic modeling strategy

**Fig. 2:**
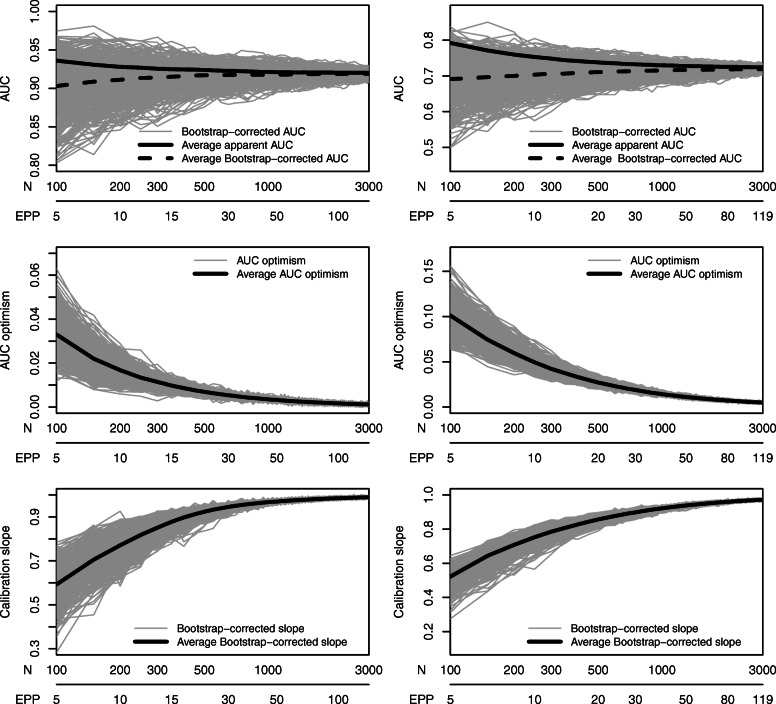
Learning curves of 500 repetitions of bootstrap-corrected AUC, AUC optimism, and bootstrap-corrected calibration slope for the ovarian cancer data (left) and the coronary artery disease data (right)

Across the 500 repetitions, there was considerable variability at low sample sizes. As expected, this variability reduced with larger sample size (Fig. 2).

The adaptive stopping rules required more patients than Riley’s initial estimate (Tables 2 and 3). Specifically, the median sample size required to observe a calibration slope ≥ 0.9 and AUC optimism ≤ 0.02 on two consecutive evaluations was 29% higher for the ovarian cancer data (*N* = 450 vs *N* = 350) and 21% higher for the CAD data (*N* = 850 vs *N* = 700). The impact of using a stopping rule with a stronger requirement for AUC optimism (≤ 0.01 instead of ≤ 0.02) depended on the case study. For the ovarian cancer data, this added 11% to the median sample size (*N* = 500 vs *N* = 450), whereas for the CAD data this added 76% (*N* = 1500 vs *N* = 850).

The method to determine sample size had impact on performance. The 10 EPP rule resulted in a median calibration slope around 0.8. Riley’s method resulted in a median slope that approached 0.9. The adaptive rules had a median slope above 0.9, in line with the stopping rules that were used. The additional requirement of an AUC optimism ≤ 0.01 resulted in higher slopes only for the CAD data.

**Fig. 3:**
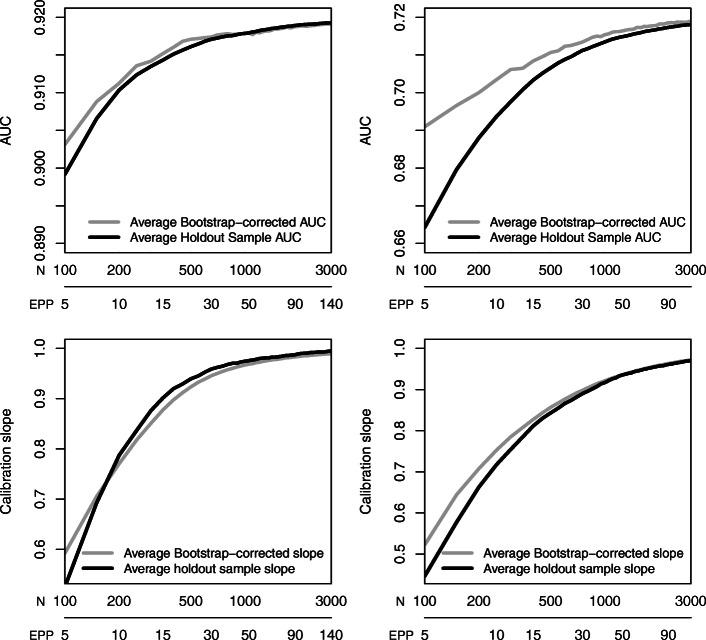
Comparison of average performance of models based on bootstrap-correction versus the use of a holdout sample for the ovarian cancer data (left) and the CAD data (right)

Holdout estimates of the AUC and calibration slope were in reasonable agreement with bootstrap-corrected estimates (Fig. 3). At low sample size, the bootstrap-corrected AUC estimates were on average higher than holdout estimates, and bootstrap-corrected calibration slopes were on average closer to 1 than holdout estimates. As sample size increased, the differences between holdout and bootstrap-corrected performance became very small.

### Evaluation of alternative modeling strategies

**Fig. 4:**
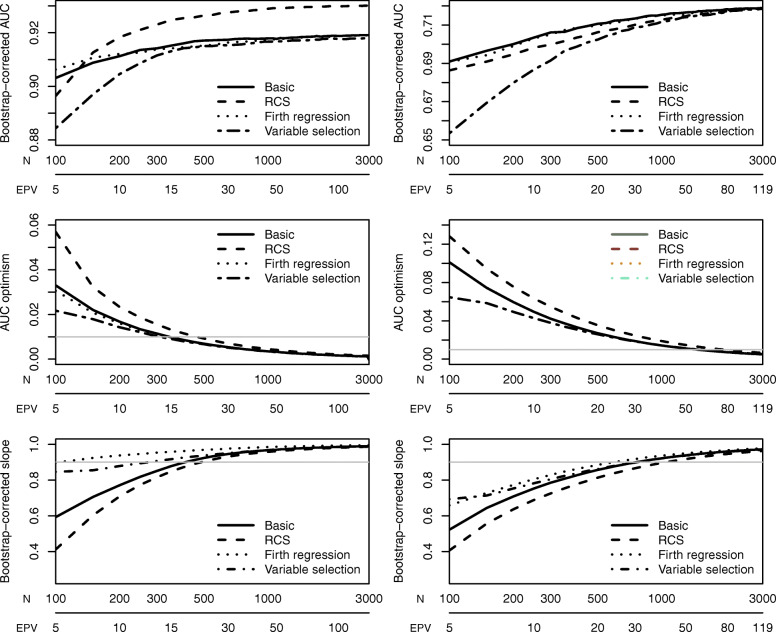
Average learning curves for all modeling strategies for the ovarian cancer data (left) and the CAD data (right)

When addressing functional form using restricted cubic splines, the number of parameters increased from 7 to 10 for the ovarian cancer case study and from 12 to 16 for the CAD case study. For 10 EPP, 307 patients are required for the ovarian cancer data (+ 43% compared to the basic strategy) and 368 for the CAD data (+ 33%). Riley’s method suggested sample sizes of at least 436 (ovarian cancer, + 39%) and 874 (CAD, + 31%) for this strategy. For the adaptive method, the median required sample size increased by 20–22% (depending on the stopping rule) for the ovarian cancer data and by 23-29% for the CAD data in comparison to the basic modeling strategy. Learning curves are shown in Figure S2, summary results in Fig. 4 and Tables 2 and 3. Hence, in the ovarian cancer data, the extra parameters for the spline functions were “cheaper” than in the CAD data. For example, for the first stopping rule 64 (450/7) patients per parameter were required for the basic strategy in the ovarian cancer data. For the three spline parameters, (550–450)/3 = 33 patients per parameter were needed, or 33*0.33 = 11 EPP. In the CAD data, the first stopping rule required 71 (850/12) patients per parameter for the basic strategy and (1100–850)/4 = 63 patients per spline parameter (or 63*0.44 = 28 EPP).

**Table 2:** Performance of the ovarian cancer models based on the determined sample size for various fixed and adaptive sample size methods. Results are shown as medians (with interquartile ranges) across 500 repetitions

	Sample size		Bootstrap-corrected performance	
Sample size method	N	EPP	AUC	Slope
***Basic strategy***				
Fixed^a^: 10 EPP	250 (250–250)	11 (11–12)	0.914 (0.901–0.926)	0.813 (0.776–0.845)
Fixed^a^: Riley’s method	350 (350–350)	16 (16–17)	0.916 (0.904–0.927)	0.884 (0.860–0.899)
Adaptive: stopping rule 1^b^	450 (450–500)	22 (20–24)	0.916 (0.907–0.926)	0.921 (0.914–0.930)
Adaptive: stopping rule 2^b^	500 (450–550)	23 (21–24)	0.918 (0.908–0.927)	0.924 (0.916–0.933)
***Restricted cubic splines***				
Fixed^a^: 10 EPP	350 (300–350)	11 (10–11)	0.925 (0.915–0.935)	0.840 (0.813–0.859)
Fixed^a^: Riley’s method	450 (450–450)	15 (14–15)	0.926 (0.917–0.935)	0.893 (0.878–0.903)
Adaptive: stopping rule 1^b^	550 (500–600)	18 (17–19)	0.928 (0.919–0.935)	0.917 (0.900–0.945)
Adaptive: stopping rule 2^b^	600 (550–600)	19 (18–20)	0.928 (0.920–0.935)	0.921 (0.914–0.927)
***Firth’s correction***				
Fixed^a^: 10 EPP	250 (200–250)	11 (10–12)	0.914 (0.897–0.929)	0.944 (0.927–0.959)
Fixed^a^: Riley’s method	350 (350–350)	16 (16–17)	0.915 (0.903–0.927)	0.958 (0.949–0.968)
Adaptive: stopping rule 1^b^	250 (200–250)	11 (10–12)	0.916 (0.901–0.930)	0.947 (0.933–0.964)
Adaptive: stopping rule 2^b^	400 (350–450)	18 (17–21)	0.916 (0.906–0.928)	0.964 (0.956–0.973)
***Including backward selection***				
Fixed^a^: 10 EPP	250 (250–250)	11 (11–12)	0.909 (0.894–0.925)	0.892 (0.875–0.907)
Fixed^a^: Riley’s method	350 (350–350)	16 (16–17)	0.913 (0.903–0.925)	0.907 (0.904–0.928)
Adaptive: stopping rule 1^b^	350 (300–400)	16 (13–18)	0.913 (0.901–0.926)	0.918 (0.910–0.926)
Adaptive: stopping rule 2^b^	400 (350–450)	18 (15–21)	0.915 (0.905–0.927)	0.926 (0.918–0.935)

*AUC* area under the receiver operating characteristic curve (or c-statistic), *slope* calibration slope, *EPP* events per parameter

^a^The analysis went in batches of 50 patients, therefore fixed sample sizes were rounded upwards to the next multiple of 50

^b^Stopping rule 1: calibration slope ≥ 0.9 and AUC optimism < = 0.02 at two consecutive assessments. Stopping rule 2: calibration slope ≥ 0.9 and AUC optimism < = 0.01 at two consecutive assessments

**Table 3:** Performance of the CAD models based on the determined sample size for various fixed and adaptive sample size methods. Results are shown as medians (with interquartile ranges) across 500 repetitions

	Sample size		Bootstrap-corrected performance	
Sample size method	***N***	EPP	AUC	Slope
***Basic strategy***				
Fixed^a^: 10 EPP	300 (250–300)	11 (10–11)	0.706 (0.684–0.726)	0.774 (0.751–0.795)
Fixed^a^: Riley’s method	700 (700–700)	28 (27–28)	0.713 (0.701–0.726)	0.894 (0.886–0.901)
Adaptive: stopping rule 1^b^	850 (750–900)	33 (30–35)	0.717 (0.705–0.727)	0.909 (0.905–0.914)
Adaptive: stopping rule 2^b^	1500 (1400–1550)	59 (56–62)	0.719 (0.712–0.725)	0.949 (0.946–0.952)
***Restricted cubic splines***				
Fixed^a^: 10 EPP	350 (350–400)	11 (10–11)	0.703 (0.684–0.721)	0.766 (0.745–0.783)
Fixed^a^: Riley’s method	900 (900–900)	26 (26–27)	0.712 (0701–0.724)	0.887 (0.877–0.894)
Adaptive: stopping rule 1^b^	1100 (1050–1200)	31 (28–33)	0.715 (0.705–0.724)	0.907 (0.904–0.911)
Adaptive: stopping rule 2^b^	1850 (1800–1900)	58 (55–60)	0.718 (0.712–0.724)	0.947 (0.945–0.949)
***Firth’s correction***				
Fixed^a^: 10 EPP	300 (250–300)	11 (10–11)	0.706 (0.682–0.726)	0.822 (0.797–0.846)
Fixed^a^: Riley’s method	700 (700–700)	28 (27–28)	0.712 (0.700–0.726)	0.913 (0.904–0.923)
Adaptive: stopping rule 1^b^	750 (700–800)	31 (28–32)	0.713 (0.703–0.727)	0.922 (0.917–0.928)
Adaptive: stopping rule 2^b^	1500 (1450–1600)	59 (56–63)	0.718 (0.710–0.726)	0.958 (0.956–0.961)
***Including backward selection***				
Fixed^a^: 10 EPP	300 (250–300)	11 (10–11)	0.690 (0.666–0.715)	0.798 (0.772–0.819)
Fixed^a^: Riley’s method	700 (700–700)	28 (27–28)	0.708 (0.694–0.722)	0.894 (0.883–0.905)
Adaptive: stopping rule 1^b^	800 (750–900)	33 (29–36)	0.712 (0.701–0.723)	0.909 (0.905–0.915)
Adaptive: stopping rule 2^b^	1550 (1400–1650)	61 (56–65)	0.716 (0.709–0.724)	0.948 (0.946–0.951)

*AUC* area under the receiver operating characteristic curve (or c-statistic), *slope* calibration slope, *EPP* events per parameter

^a^The analysis went in batches of 50 patients, therefore fixed sample sizes were rounded upwards to the next multiple of 50

^b^Stopping rule 1: calibration slope ≥ 0.9 and AUC optimism < = 0.02 at two consecutive assessments. Stopping rule 2: calibration slope ≥ 0.9 and AUC optimism < = 0.01 at two consecutive assessments

The use of Firth’s correction led to a lower required sample size (Figure S3, Fig. 4, Tables 2 and 3) compared to the basic modeling strategy. However, we observed differences between the two case studies, with a larger reduction for the ovarian cancer data than for the CAD data. The sample size reduction was larger for the first stopping rule than for the second stopping rule. Of note, the median sample size decreased by 44% for the first stopping rule in the ovarian cancer study (250 vs 450), but did not change for the second stopping rule in the CAD study (1500 vs 1500). On average, the model performance improved with the use of Firth’s correction.

The inclusion of backward variable elimination resulted in similar or lower required sample sizes compared with the basic strategy (Figures S4–5, Fig. 4, Tables 2 and 3). In the ovarian cancer data, the median sample size decreased by 22% for the first stopping rule (350 vs 450) and by 20% for the second stopping rule (400 vs 500). In the CAD data, the median sample size decreased by 6% for the first stopping rule (800 vs 850) and increased by 3% for the second stopping rule (1550 vs 1500). Figures S6–S7 present the selection proportion of each predictor at each sample size update for both case studies.

For alternative modeling strategies, we again observed that bootstrap-corrected performance was typically higher than holdout performance at low sample size, but that differences quickly became very small with increasing sample size (Figures S8–S10).

## Discussion

The proposed adaptive sample size determination procedure is specific for the development of a clinical prediction model in the modeling and data context at hand. Our adaptive stopping rules led to much higher sample sizes than the 10 EPP rule, even more than 20 EPP was needed. These results are consistent with the finding that EPP requirements increase with the event fraction [8]. The required sample size was also slightly larger than when using the fixed calculation method by Riley and colleagues [6]. The choice of both modeling strategy and the specific stopping rule had impact on the required sample size, but the impact depended on the modeling context. We observed considerable variability in model performance, particularly at low sample sizes. Perhaps surprisingly, the inclusion of variable selection reduced the sample size for the ovarian cancer data. This may be caused by strong preselection of predictors (Figure S6) and by the relationship between the maximum diameters of the lesion and the largest solid component. These diameters are clearly correlated, with the latter diameter bounded by the former. The variable selection typically excluded the maximum lesion diameter.

The adaptive sample size procedure monitors model performance during data collection. The main strength of the adaptive procedure is that it is able to incorporate more complex modeling scenarios than the existing methods for sample size estimation. It can for example account for imputation of missing data, modeling of nonlinear relations, variable selection, and penalization algorithms. Thus, one can tailor the estimate of the required sample size to the specific modeling context. Moreover, our method can nicely complement Riley’s a priori fixed calculation method. We recommend to provide a reasonable estimate of the minimum sample size upfront (*N*_0_ in the adaptive procedure above), so that the feasibility of collecting this amount of data is ensured. Riley’s method is an important tool to do so. Then, the adaptive approach can be used to adjust the initial estimate if needed. Whereas this upfront calculation focuses on the minimal sample size at which desired performance can be expected, adaptive sample size monitoring can help to find the sample size at which there is empirical support for the desired performance.

The adaptive procedure requires several parameters to be set, such as *N*_start_, *N*_add_, and the stopping rules. These values can be chosen depending on the situation, the key issue being that the choices should be transparent. To set *N*_start_ and *N*_add_, arguments such as *N*_0_, the anticipated or even the actual recruitment rate, and the effort needed to prepare data for analysis can be used. Other stopping rules than the ones we have used can be derived. The calibration slope directly focuses on overfitting in terms of the accuracy of the estimated risks and may therefore be seen as perhaps the most important measure. AUC optimism is also useful regarding overfitting, but, as the case studies also indicated, smaller AUC optimism is easier to obtain when the true underlying AUC value is high. As overall measures of performance, Brier score or R-squared may be used to define stopping rules as well [6, 18].

Analogous to the use of interim analyses in randomized trials, halting recruitment because a stopping rule has been met bears a risk of stopping too early, leading to models with lower performance on new data from the same population. In our view, this risk can be controlled with the following measures. First, the modeling strategy and preferably also the stopping rule should be specified in advance, and the learning curves should be reported. Second, it makes sense to perform a reasonable a priori fixed sample size calculation to have an indication of the approximate minimum sample size. Third, it is sensible to include a requirement that the target performance (e.g. in terms of AUC optimism and calibration slope) be met on more than one consecutive assessment. The specifics of this may depend on the chosen value of *N*_add_; the larger *N*_add_, the lower the need for such a requirement may be. A true steady state implies that the calibration slope approaches 1 and optimism in AUC approaches 0. As long as resources permit, it is advisable to continue increasing the sample size even when the stopping rules have been met. The learning curves are helpful in providing such insight.

Apart from application in prospective studies, this procedure can also be applied to retrospective studies on existing patient cohorts. Preparing data from existing cohorts for prediction modeling is not always straightforward, for example when biomarkers have to be quantified on available blood samples or when extensive data cleaning is required. The adaptive sample size procedure can then be applied to know how many cases have to be prepared. For retrospective applications, cases should be added in reverse chronological order: first the most recent *N*_start_ cases, and then work backwards *N*_add_ cases at a time. This avoids that the most recent available data are not used in the end.

A limitation of the adaptive procedure is that the final sample size is not set in advance, which may lead to practical and logistical shortcomings. For example, more data cleaning and computational efforts are required, and studies may take longer to complete if the stopping rule is met at a higher sample size than anticipated. On the other hand, although using a fixed sample size does not have this drawback, it is uncertain how reasonable the fixed sample size turns out to be in the end. Another consequence of our procedure is that, for prospective studies, continuous data monitoring and data cleaning is required. This additional effort is probably more an advantage than a limitation, because continuous evaluation of incoming data tends to save time later on and can timely spot and remedy any data collection issues. Finally, the adaptive procedure is most attractive for settings where outcomes are immediately known (diagnostic research) or within a short period of follow-up (e.g., complications after surgery or 30-day mortality).

A limitation of the resampling study may be that we sampled from the datasets without replacement rather than with replacement. We deliberately opted to sample without replacement to mimic real-life recruitment. However, this may have led to an underestimation of the variability between learning curves (Figures S11–12).

Future research should focus on learning curves to further study how the required sample size is impacted by contextual characteristics such as modeling choices (type of algorithm, amount of a priori and data-driven variable selection), case mix (distribution of predictors and outcomes), and predictive strength. Although this was not addressed systematically in this work, predictive strength of the included predictors, as expressed by the AUC, plays a role. The ovarian cancer (AUC around 0.9) and CAD case study (AUC around 0.7) are clearly different in this respect.

## Conclusions

Adaptive sample size determination can play an important role to obtain a context-specific estimate of the sample size that is required for developing a robust prediction model. Sample size determination for the development of a clinical risk prediction model can combine an a priori fixed calculation with the suggested adaptive procedure to empirically monitor and determine the final sample size.

## Supplementary Information

**Additional file 1: Supplementary material.**

## Data Availability

For the CAD data, collaboration is welcomed and data sharing can be agreed upon by contacting Michael Edlinger (michael.edlinger@i-med.ac.at). The ovarian cancer dataset can be made available on reasonable request from Dirk Timmerman (dirk.timmerman@uzleuven.be).

## Abbreviations

EPP: Events per predictor parameter
EPV: Events per variable
AUC: Area under the receiver operating characteristic curve
RCS: Restricted cubic splines
CAD: Coronary artery disease
