# Supplementary material for: Adaptive sample size determination for the development of clinical prediction models
Source: Diagn Progn Res. 2021 Mar 22;5:6. doi: 10.1186/s41512-021-00096-5 (PMC7983402; doi:10.1186/s41512-021-00096-5)
Supplement: Supplementary file 1 — Additional file 1: Supplementary material. [file 41512_2021_96_MOESM1_ESM.docx]

Supplementary Material

Adaptive sample size determination for the development of risk prediction models

Evangelia Christodoulou^1^, Maarten van Smeden^2^, Michael Edlinger^1,3^, Dirk Timmerman^1,4^, Maria Wanitschek^5^, Ewout W Steyerberg^6^, Ben Van Calster^1, 6, 7^

^1^ Department of Development & Regeneration, KU Leuven, Leuven, Belgium. ^2^ University Medical Center Utrecht, Utrecht, Netherlands.^3^ Department of Medical Statistics, Informatics, and Health Economics, Medical University Innsbruck, Austria. ^4^ University Hospitals Leuven, Leuven, Belgium. ^5^ University Clinic of Internal Medicine III - Cardiology and Angiology, Tirol Kliniken, Innsbruck, Austria. ^6^ Department of Biomedical Data Sciences, Leiden University Medical Center, Leiden, Netherlands. ^7^ EPI-centre, KU Leuven, Leuven, Belgium

## **Appendix A. Learning Curves Methodology for the Combination of Multiple Imputation with bootstrapping**

The adaptive procedure goes as follows (based on the on the Val-MI procedure outlined in Wahl et al^1^) :

1. Determine an initial estimation of the required sample size (N_0_). This is best done using the recently suggested fixed sample size determination procedure from Riley and colleagues, based on the number of candidate parameters, the assumed outcome event fraction, and the anticipated Cox-Snell R-squared.
2. Determine a sample size N_start_ (<N_0_) at which performance is estimated for the first time, and recruit N_start_ patients in the study. This is the first model development dataset.
3. Use an imputation algorithm (e.g. the method of fully chained equations using the mice package in R software) to generate M multiply imputed (completed) development datasets.
4. Apply the prespecified modeling strategy on the M completed development datasets, average the coefficients of the M models in order to obtain model M_D_.
5. Fit M_D_ on each of the M completed development datasets and evaluate performance. Calculate the average performance and store this as the apparent performance (PM_D_; i.e. performance on exactly the same data that were used to obtain the model).
6. Perform internal validation; we use Harrell’s enhanced bootstrap, a recommended method that has been shown to perform well.^2–4^ In the presence of multiple imputation, we apply the enhanced bootstrap as follows:
   1. draw a bootstrap sample with replacement from the development dataset
   2. use the imputation algorithm from iii to generate M multiply imputed (completed) bootstrap datasets
   3. apply the modeling strategy on each of the M completed bootstrap datasets, and average model coefficients to obtain M_B_
   4. fit the model with the average coefficients on each of the M completed bootstrap datasets and average the performance measures (PM_B_);
   5. fit the model with the average coefficients on each of the M completed original datasets, and average the performance measures (PM_O_)
   6. calculate the optimism as the difference PM_B_-PM_O_ for each performance measure
   7. repeat a.-f. B-1 times, and calculate the average of the B optimism estimates
   8. subtract the average optimism from the apparent performance PM_D_ to obtain internally validated performance estimates for model M_D_–denoted as "bootstrap-corrected" performance estimates in this study
7. Recruit N_add_ new patients, add them to the development dataset, and repeat steps iii.- vi.
8. Repeat step vii. until a prespecified stopping rule has been reached; the model M_D_ for which the stopping rule has been reached is the final prediction model

In our resampling study, we sampled without replacement from the available datasets, with N_start_ = 100, M = 20, B = 200, and N_add_ = 50. We continued until a sample size of 3000 was reached, even when the stopping rule was reached earlier.

Because this procedure is computationally intensive, this process was performed only once. Bootstrap validation involved fitting 20 models in each of 200 iterations, i.e. 4000 models in total. In practical applications, we anticipate this process can be made computationally more efficient by reducing the number of imputations and/or the number of iterations, but we did not investigate this.

## **Appendix B. Handling of warnings and errors**

#### Recording and handling of warnings/errors

Errors and warnings relate to possible separation of the model fit or to non-variability in binary predictors, and occurred when we fit the model on the training set or on bootstrap datasets during internal validation. Initially, the plan was to start with a training set of 50 patients. Due to the high number of errors and warnings that occurred related to the lack of variability of binary predictors or possible separation of the logistic regression model, we decided to start with training sets of 100 patients.

For model fitting on the training set, we report per sample size update the percentage of training sets across the 500 repetitions in which a warning/error message was given. For bootstrapping, we report per sample size update the percentage of bootstrap samples (across the 500 (repetitions) x 200 (bootstrap samples in each repetition = 100000 samples) in which a warning/error was given.

The datasets in which a warning for possible separation was given were kept in our analysis. Datasets with non-variability of a binary predictor or another error only occurred in bootstrap samples and were excluded. Moreover, the further error messages that were encountered in the bootstrap samples of the variable selection strategy were also excluded.

*Table B.1. Ovarian cancer data: Percentage of model fits with errors/warnings for the basic modeling strategy*

|  | **Sample size** | | | | | | | | | | |  |
| --- | --- | --- | --- | --- | --- | --- | --- | --- | --- | --- | --- | --- |
| **Error/warning** | **100** | **150** | **200** | **250** | **300** | **350** | **400** | **450** | **500** | **550** | **600** |  |
| Fit model on training data (over 500 model fits) | | | | | | | | | | | | |
| Separation suggested^1^ | 14.2 | 5.6 | 2.8 | 1.0 | 0.8 | 0.4 | 0 | 0 | 0 | 0 | 0 |  |
| Fit model on bootstrap data (over 500x200 bootstrap model fits) | | | | | | | | | | | | |
| Separation suggested^1^ | 28 | 15 | 9.3 | 5.7 | 3.6 | 2.1 | 1.4 | 1.0 | 0.5 | 0.4 | 0.3 |  |
| No variability in predictor^2^ | 26 | 0.5 | 0 | 0 | 0 | 0 | 0 | 0 | 0 | 0 | 0 |  |

^1^ Separation warning (R message: “glm.fit: fitted probabilities numerically 0 or 1 occurred”)

^2^ No variability in binary predictor (R message: “contrasts can be applied only to factors with 2 or more levels”)

*Table B.2. Ovarian cancer data: Percentage of model fits with errors/warnings for the RCS modeling strategy*

|  | **Sample size** | | | | | | | | | | |
| --- | --- | --- | --- | --- | --- | --- | --- | --- | --- | --- | --- |
| **Error/warning** | **100** | **150** | **200** | **250** | **300** | **350** | **400** | **450** | **500** | **550** | **600** |
| Fit model on training data (over 500 model fits) | | | | | | | | | | | |
| Separation suggested ^1^ | 17.8 | 4.2 | 1.0 | 0.4 | 0.2 | 0 | 0 | 0 | 0 | 0 | 0 |
| Fit model on bootstrap data (over 500x200 bootstrap model fits) | | | | | | | | | | | |
| Separation suggested^1^ | 49.7 | 21.6 | 10.0 | 5.0 | 2.7 | 1.3 | 0.7 | 0.4 | 0.2 | 0.1 | 0 |
| No variability in predictor^2^ | 19.0 | 1 | 0 | 0 | 0 | 0 | 0 | 0 | 0 | 0 | 0 |

^1^ Separation warning (R message: “glm.fit: fitted probabilities numerically 0 or 1 occurred”)

^2^ No variability in binary predictor (R message: “contrasts can be applied only to factors with 2 or more levels”)

*Table B.3. Ovarian cancer data: Percentage of model fits with errors/warnings for the Firth regression modeling strategy*

|  | **Sample size** | | | | | | | | | | |
| --- | --- | --- | --- | --- | --- | --- | --- | --- | --- | --- | --- |
| **Error/warning** | **100** | **150** | **200** | **250** | **300** | **350** | **400** | **450** | **500** | **550** | **600** |
| Fit model on training data (over 500 model fits) | | | | | | | | | | | |
| Separation suggested^1^ | 0 | 0 | 0 | 0 | 0 | 0 | 0 | 0 | 0 | 0 | 0 |
| Fit model on bootstrap data (over 500x200 bootstrap model fits) | | | | | | | | | | | |
| Separation suggested^1^ | 4.4 | 1.6 | 0.4 | 0.3 | 0.1 | 0.06 | 0 | 0 | 0 | 0 | 0 |
| No variability in predictor^2^ | 14.5 | 0 | 0 | 0 | 0 | 0 | 0 | 0 | 0 | 0 | 0 |

^1^ Separation warning (R message: “glm.fit: fitted probabilities numerically 0 or 1 occurred”)

^2^ No variability in binary predictor (R message: “contrasts can be applied only to factors with 2 or more levels”)

*Table B.4. Ovarian cancer data: Percentage of model fits with errors/warnings for the variable selection strategy*

|  | **Sample size** | | | | | | | | | | |  |
| --- | --- | --- | --- | --- | --- | --- | --- | --- | --- | --- | --- | --- |
| **Error/warning** | **100** | **150** | **200** | **250** | **300** | **350** | **400** | **450** | **500** | **550** | **600** |  |
| Fit model on training data (over 500 model fits) | | | | | | | | | | | | |
| Separation suggested ^1^ | 0.02 | 0 | 0 | 0 | 0 | 0 | 0 | 0 | 0 | 0 | 0 |  |
| Fit model on bootstrap data (over 500x200 bootstrap model fits) | | | | | | | | | | | | |
| Separation suggested ^1^ | 2.9 | 0.2 | 0.02 | 0 | 0 | 0 | 0 | 0 | 0 | 0 |  |  |
| Extra error messages^3^ | 14.5 | 1.0 | 0.09 | 0.03 | 0.02 | 0.02 | 0.01 | 0.01 | 0 | 0 | 0 |  |

^1^ Separation warning (R message: “glm.fit: fitted probabilities numerically 0 or 1 occurred”)

^3^ Fit does not have design information (stemming from fastbw function R error message: “fit does not have design information") & argument of length zero (stemming from fastbw function R error message: “argument of length zero ")

*Table B.5. CAD data: Percentage of model fits with errors/warnings for the basic modeling strategy*

|  | **Sample size** | | | | | | | | | | |
| --- | --- | --- | --- | --- | --- | --- | --- | --- | --- | --- | --- |
| **Error/warning** | **100** | **150** | **200** | **250** | **300** | **350** | **400** | **450** | **500** | **550** | **600** |
| Fit model on training data (over 500 model fits) | | | | | | | | | | | |
| Separation suggested ^1^ | 0 | 0 | 0 | 0 | 0 | 0 | 0 | 0 | 0 | 0 | 0 |
| Fit model on bootstrap data (over 500x200 bootstrap model fits) | | | | | | | | | | | |
| Separation suggested ^1^ | 0.4 | 0 | 0 | 0 | 0 | 0 | 0 | 0 | 0 | 0 | 0 |
| No variability in predictor^2^ | 0.1 | 0 | 0 | 0 | 0 | 0 | 0 | 0 | 0 | 0 | 0 |

^1^ Separation warning (R message: “glm.fit: fitted probabilities numerically 0 or 1 occurred”)

^2^ No variability in binary predictor (R message: “contrasts can be applied only to factors with 2 or more levels”)

*Table B.6. CAD data: Percentage of model fits with errors/warnings for the RCS modeling strategy*

|  | **Sample size** | | | | | | | | | | | |  |
| --- | --- | --- | --- | --- | --- | --- | --- | --- | --- | --- | --- | --- | --- |
| **Error/warning** | **100** | **150** | **200** | **250** | **300** | **350** | **400** | **450** | **500** | **550** | **600** |  |  |
| Fit model on training data (over 500 model fits) | | | | | | | | | | | | | |
| Separation suggested ^1^ | 0 | 0 | 0 | 0 | 0 | 0 | 0 | 0 | 0 | 0 | 0 |  |  |
| Fit model on bootstrap data (over 500x200 bootstrap model fits) | | | | | | | | | | | | | |
| Separation suggested ^1^ | 2.9 | 0.04 | 0 | 0 | 0 | 0 | 0 | 0 | 0 | 0 | 0 |  |  |
| No variability in predictor^2^ | 0.01 | 0 | 0 | 0 | 0 | 0 | 0 | 0 | 0 | 0 | 0 |  |  |

^1^ Separation warning (R message: “glm.fit: fitted probabilities numerically 0 or 1 occurred”)

^2^ No variability in binary predictor (R message: “contrasts can be applied only to factors with 2 or more levels”)

*Table B.7. CAD data: Percentage of model fits with errors/warnings for the Firth regression modeling strategy*

|  | **Sample size** | | | | | | | | | | |  |
| --- | --- | --- | --- | --- | --- | --- | --- | --- | --- | --- | --- | --- |
| **Error/warning** | **100** | **150** | **200** | **250** | **300** | **350** | **400** | **450** | **500** | **550** | **600** |  |
| Fit model on training data (over 500 model fits) | | | | | | | | | | | | |
| Separation suggested ^1^ | 0 | 0 | 0 | 0 | 0 | 0 | 0 | 0 | 0 | 0 | 0 |  |
| Fit model on bootstrap data (over 500x200 bootstrap model fits) | | | | | | | | | | | | |
| Separation suggested ^1^ | 0.03 | 0 | 0 | 0 | 0 | 0 | 0 | 0 | 0 | 0 | 0 |  |
| No variability in predictor^2^ | 0.01 | 0 | 0 | 0 | 0 | 0 | 0 | 0 | 0 | 0 | 0 |  |

^1^ Separation warning (R message: “glm.fit: fitted probabilities numerically 0 or 1 occurred”)

^2^ No variability in binary predictor (R message: “contrasts can be applied only to factors with 2 or more levels”)

*Table B.8. CAD data: Percentage of model fits with errors/warnings for the variable selection strategy*

|  | **Sample size** | | | | | | | | | | |  |
| --- | --- | --- | --- | --- | --- | --- | --- | --- | --- | --- | --- | --- |
| **Error/warning** | **100** | **150** | **200** | **250** | **300** | **350** | **400** | **450** | **500** | **550** | **600** |  |
| Fit model on training data (over 500 model fits) | | | | | | | | | | | | |
| Separation suggested ^1^ | 0 | 0 | 0 | 0 | 0 | 0 | 0 | 0 | 0 | 0 | 0 |  |
| Fit model on bootstrap data (over 500x200 bootstrap model fits) | | | | | | | | | | | | |
| Separation suggested ^1^ | 0.3 | 0 | 0 | 0 | 0 | 0 | 0 | 0 | 0 | 0 | 0 |  |
| No variability in predictor^2^ | 0 | 0 | 0 | 0 | 0 | 0 | 0 | 0 | 0 | 0 | 0 |  |
| Extra error messages^3^ | 5.4 | 0 | 0 | 0 | 0 | 0 | 0 | 0 | 0 | 0 | 0 |  |

^1^ Separation warning (R message: “glm.fit: fitted probabilities numerically 0 or 1 occurred”)

^2^ No variability in binary predictor (R message: “contrasts can be applied only to factors with 2 or more levels”)

^3^ Fit does not have design information (stemming from fastbw function R message: “fit does not have design information")

## **Appendix C. Details of the sample size determination based on Riley’s method** ^5^

We followed the four-step procedure for the sample size determination for the development of a clinical risk prediction model according to the method of Riley et al.^5^ Eventually, the sample size that is required, is the largest sample size amongst the four steps. The calculation is based on information that was available in the literature prior to collecting the datasets.

1. **Ovarian cancer dataset**

The calculation requires the following information: number of predictive parameters, prevalence of the outcome, and an estimate of the Cox-Snell R-squared. To estimate the prevalence and Cox-Snell R-squared, we used results from Timmerman et al, a paper that presented a logistic regression model just before data collection started for the dataset that we used in our case study.^6^ This paper uses data from 191 patients, of which 51 (26.7%) were malignant. In this study, a logistic regression model that was developed on the training data had a c-statistic of 0.90 on the 57 patients in the test set. This information (c 0.9 and event fraction 0.267) was used to simulate a large dataset (N = 1 million). The outcome Y was simulated under a true event fraction of 0.267, and a predictor X that has a normal distribution in events and in non-events. In non-events, a standard normal distribution was assumed. In events, the normal distribution has mean μ and variance 1, with μ equal to $\Phi^{-1}\left( 0.9 \right)*\sqrt{2}$. $\Phi^{-1}$ is the inverse standard normal cumulative distribution function. Calculating the mean in this manner guarantees a true c-statistic of 0.9.^7^ In the simulated dataset, we fit a standard logistic regression model of Y on X, and calculate the Cox-Snell R-squared of the model. This leads to an estimate of the Cox-Snell R-squared of 0.38.

*Logistic regression with 7 predictive parameters*

Assuming a prevalence of malignancy of 26.7%, and a Cox-Snell R-squared of 0.38, we proceed with the calculation of each step of the procedure:

**Step 1** The required minimum sample size for precise estimation of model’s intercept (margin of error ≤0.05) is 301 patients.

**Step 2** The required minimum sample size for a small mean absolute prediction error (MAPE target 0.05) across all individuals is 314.

**Step 3** The required minimum sample size that will produce a small required shrinkage of predictor effects (≤10%) is 128.

**Step 4** The required minimum sample size to obtain an expected optimism of 0.05 in Nagelkerke’s R-squared is 158 patients.

Steps 1, 3, and 4 are based on the pmsampsize R package. Step 2 was based on the formula in Figure 2 of Riley et al.^5^ Based on these calculations, at least 314 patients are required. A more conservative estimate, in which we use an anticipated Cox-Snell R-squared of 0.30 also leads to 314 patients.

*Using restricted cubic splines for continuous predictors: 10 predictive parameters*

Applying the same method for 10 parameters, a prevalence of 0.267, and a Cox-Snell R-squared of 0.38, we end up with a sample size of at least 436. A more conservative estimate based on a Cox-Snell R-squared of 0.30 also leads to 436 patients.

*Conclusion*

We concluded that at least 314 and 436 patients are required for the models with 7 and 10 parameters respectively. In our analysis, we add patients in batches of 50, therefore, we rounded these numbers upwards to 350 and 450, respectively.

1. **CAD dataset**

For this case study, we based the sample size calculation on information in Genders et al,^8^ a study presenting a logistic regression model for diagnosing CAD. The CAD dataset that we used as a case study was based on this model. This paper contains data from 18 hospitals, of which 10 are from a high prevalence setting and 8 are from a low prevalence setting. Their aim was to develop a model for a low prevalence setting. The model development included all 18 hospitals, with a setting variable (low vs high). Internal validation using cross-validation focused on the low prevalence settings. The sample size was 5677, of which 1251 in low prevalence settings. The prevalence of CAD in low prevalence settings was 46% (579/1251). The cross-validated c-statistic of the model was 0.79. Using a similar approach as for the ovarian cancer data, we obtain an estimated Cox-Snell R-squared estimate of 0.24. An adjusted version of the R-squared is not strictly needed because the model was validated using cross-validation.

*Basic strategy: maximum likelihood logistic regression with 12 predictive parameters*

Assuming an outcome prevalence of 46%, and a Cox-Snell R-squared of 0.24, we proceed with the calculation of each step of the procedure:

**Step 1** The required minimum sample size for precise estimation of model’s intercept (margin of error ≤0.05) is 382 patients.

**Step 2** The required minimum sample size for a small mean absolute prediction error (MAPE target 0.05) across all individuals is 669.

**Step 3** The required minimum sample size that will produce a small required shrinkage of predictor effects (≤10%) is 387.

**Step 4** The required minimum sample size to obtain an expected optimism of 0.05 in Nagelkerke’s R-squared is 274 patients.

Based on these calculations, at least 669 patients are required.

*Using restricted cubic splines for continuous predictors: 16 predictive parameters*

Applying the same method for 16 parameters, a prevalence of 0.46, and a Cox-Snell R-squared of 0.24, we end up with a required sample size of at least 874.

*Conclusion*

We concluded that at least 669 and 874 patients are required for the models with 12 and 16 parameters respectively. In our analysis, we add patients in batches of 50, therefore, we rounded these numbers upwards to 700 and 900, respectively.

## **Appendix D. Additional Figures**

**Figure S1**. Single learning curves of bootstrap-corrected AUC, AUC optimism, and bootstrap-corrected calibration slope for the CAD dataset when missing data are handled with multiple imputation.


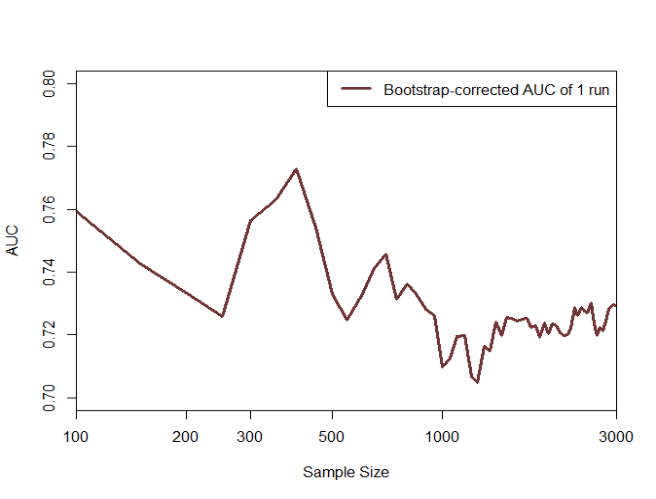

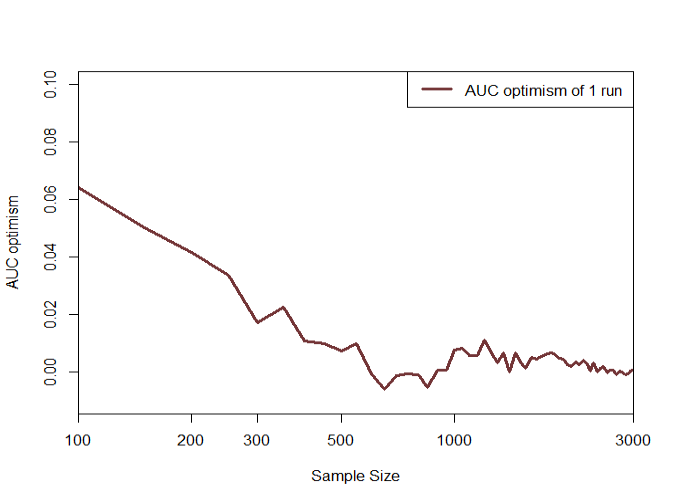


**
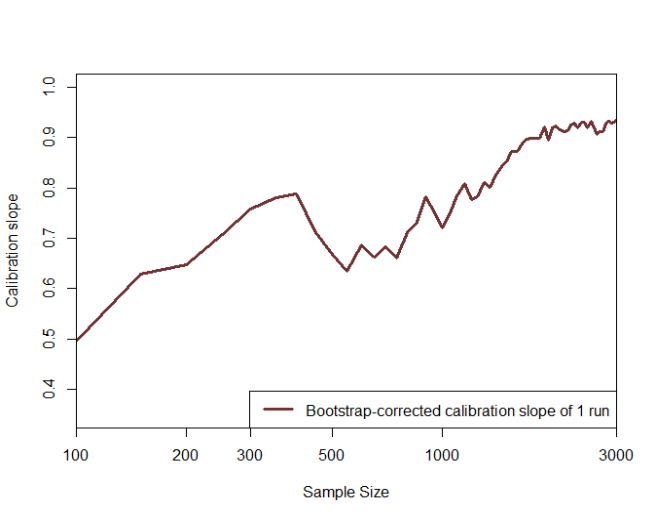
**

**Figure S2***.* Learning curves of 500 repetitions of bootstrap-corrected AUC, AUC optimism, and bootstrap-corrected calibration slope for the ovarian cancer data (left) and the CAD (right) when using the modeling strategy involving restricted cubic splines for continuous predictors.


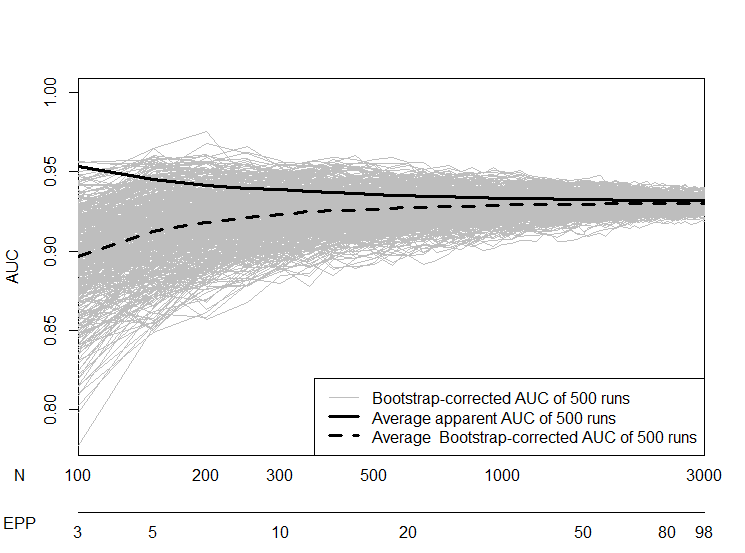

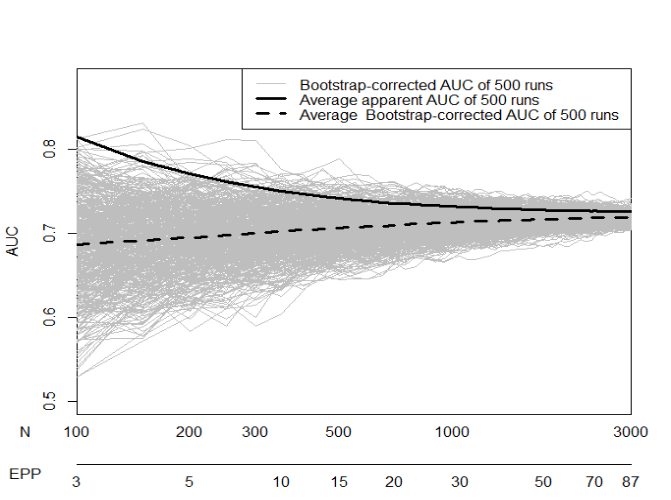


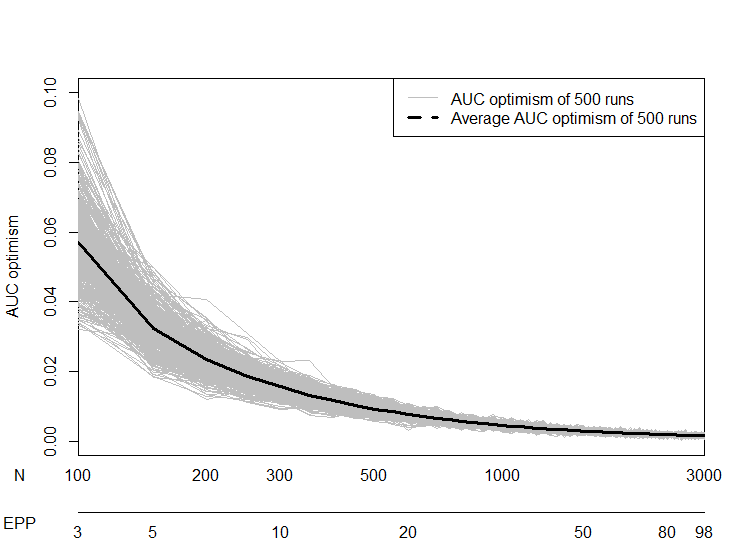

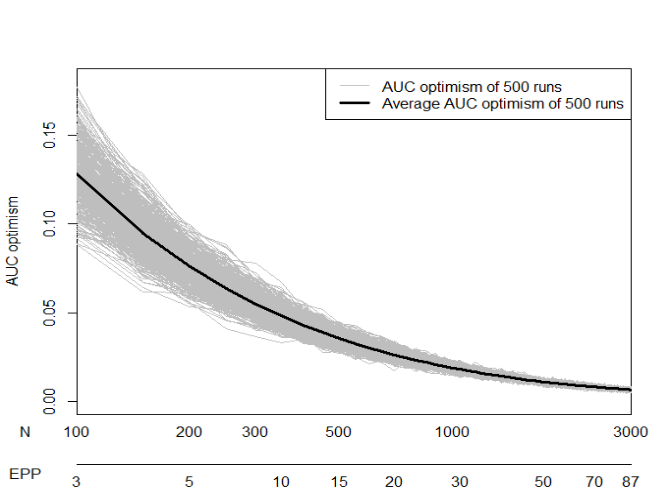


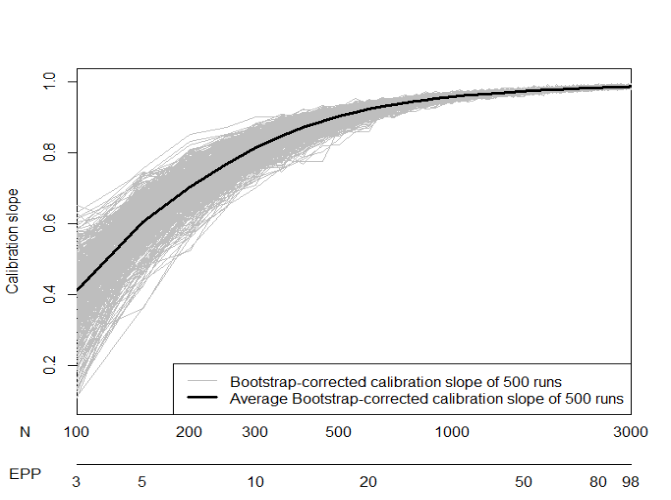

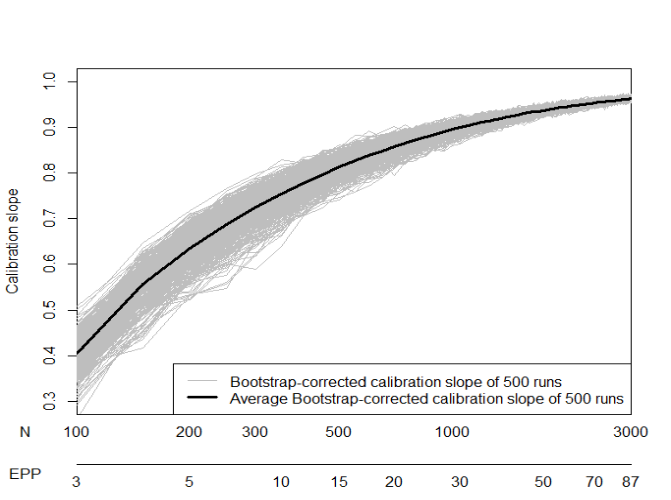


**Figure S3**. Learning curves of 500 repetitions of bootstrap-corrected AUC, AUC optimism, and bootstrap-corrected calibration for the ovarian cancer data (left) and the CAD (right) when using the modeling strategy involving Firth’s correction instead of maximum likelihood logistic regression.


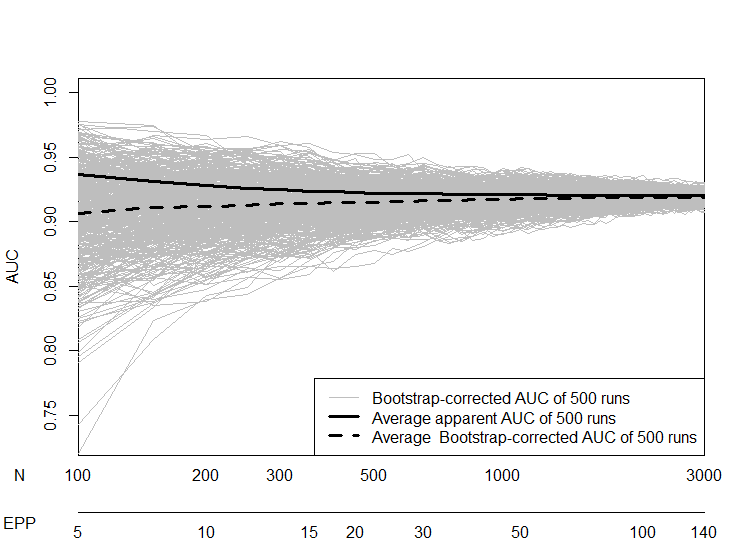

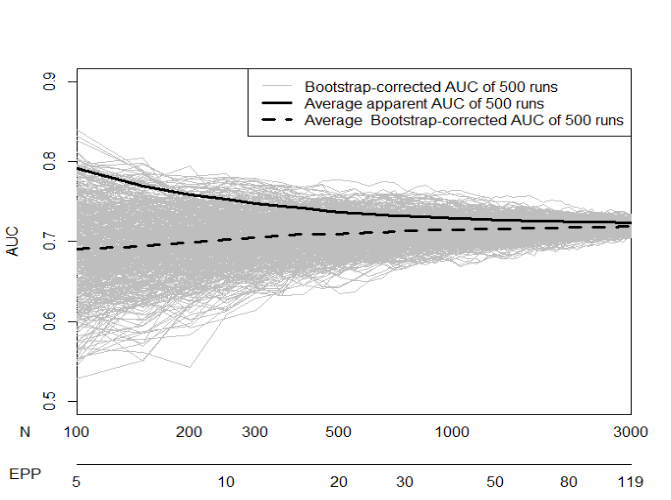


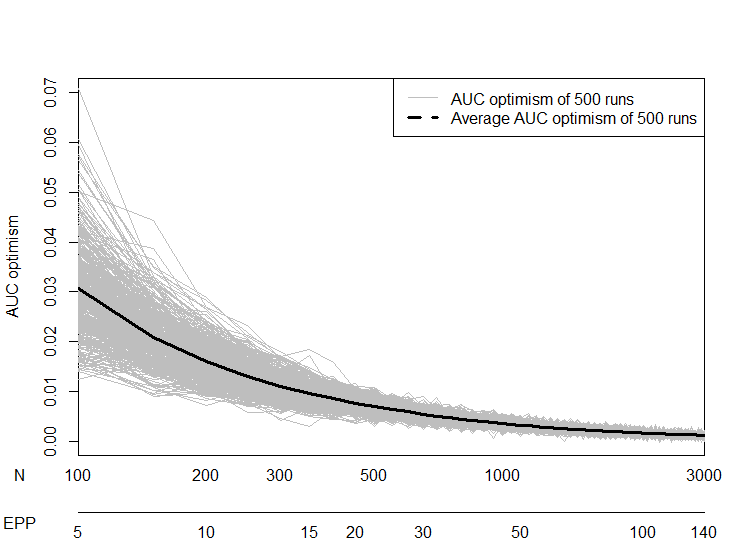

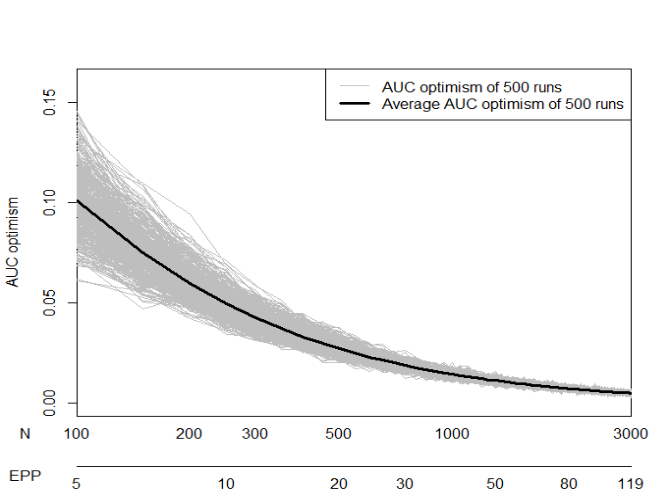


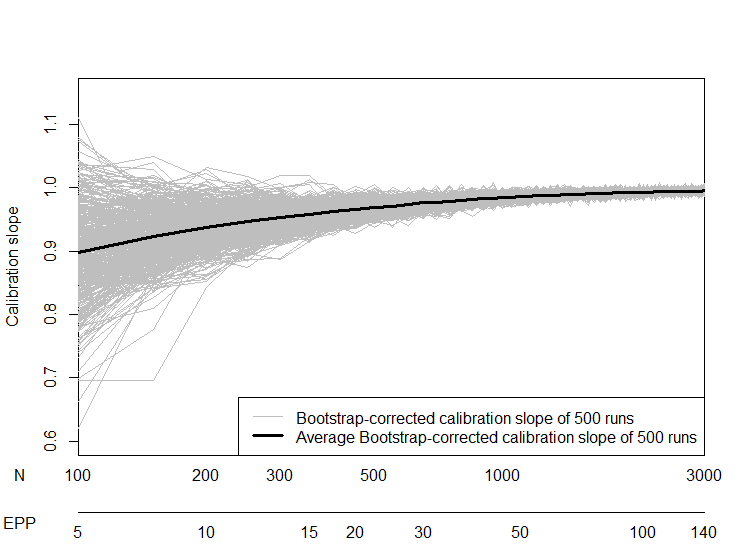

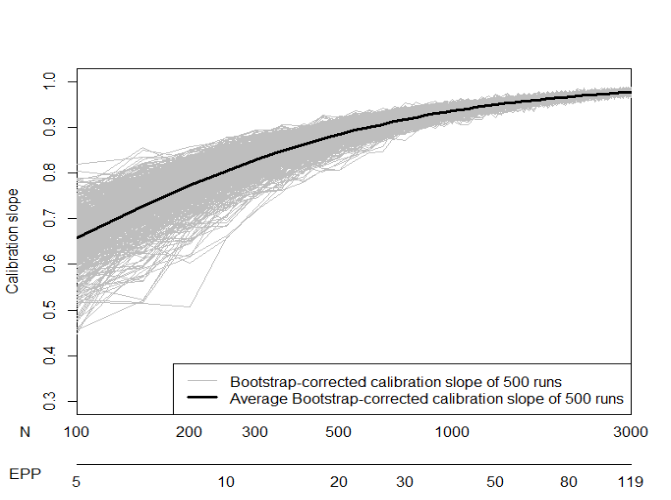


**Figure S4**. Learning curves of 500 repetitions of bootstrap-corrected AUC, AUC optimism, and bootstrap-corrected calibration slope for the ovarian cancer data when using the modeling strategy involving backward elimination.


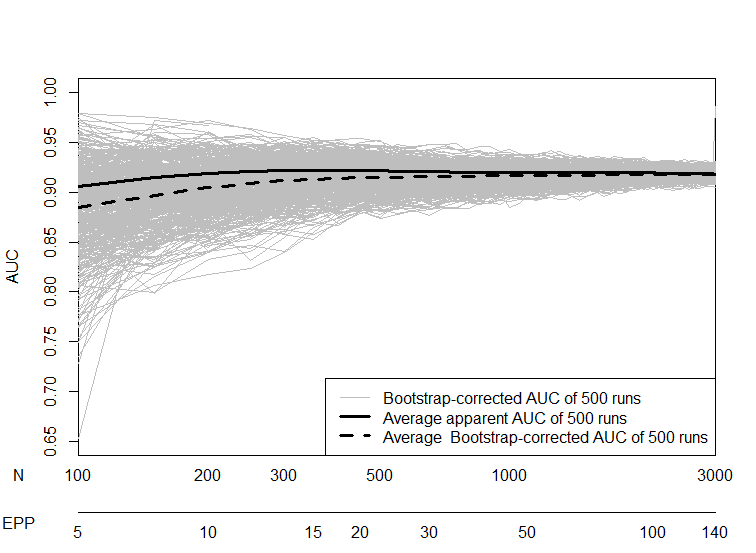

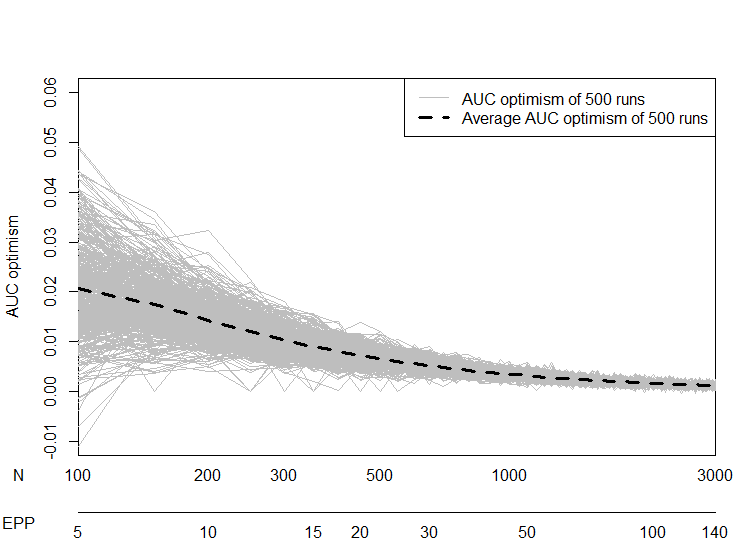


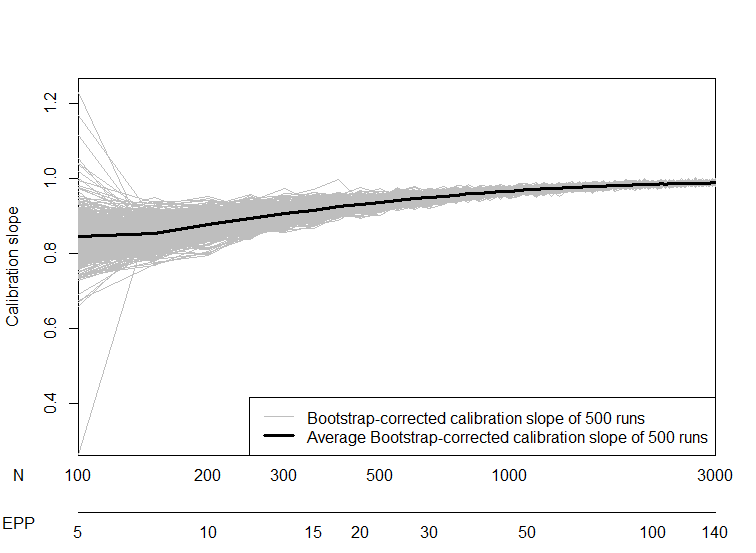


**Figure S5**. Learning curves of 500 repetitions of bootstrap-corrected AUC, AUC optimism, and bootstrap-corrected calibration slope for the CAD when using the modeling strategy involving backward elimination.


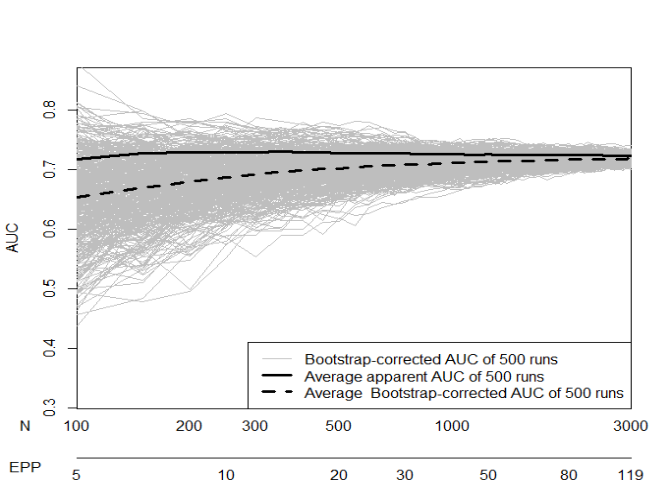

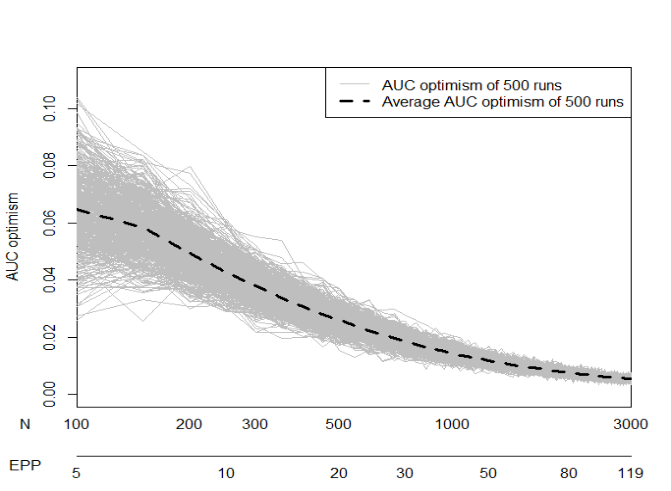


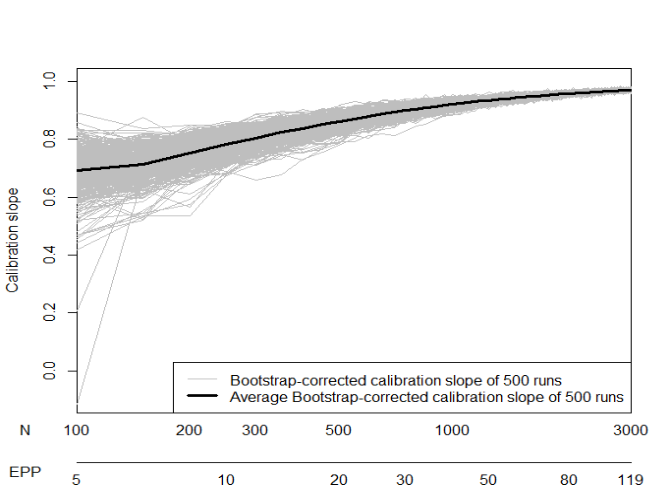


**Figure S6**. Heatmap with selection proportion (across 500 repetitions) of each predictor during backward variable elimination at every sample size update for the ovarian cancer data. Age was forced in the model.


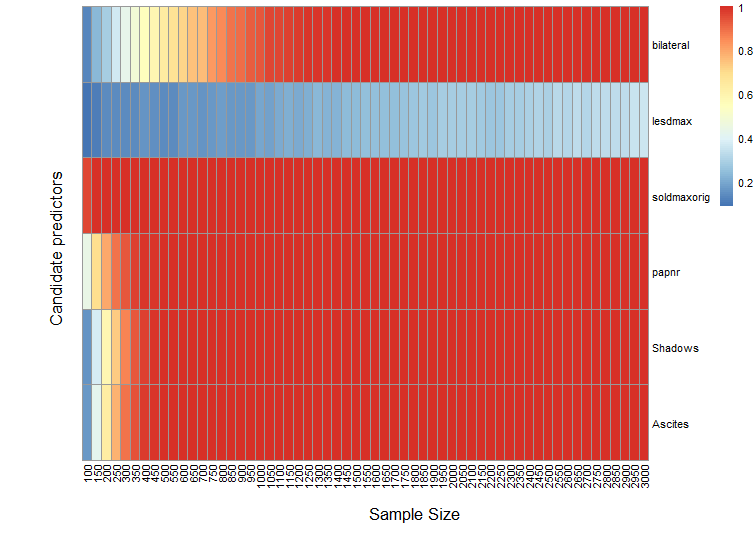


**Figure S7**. Heatmap with selection proportion (across 500 repetitions) of each predictor during backward variable elimination at every sample size update for the CAD data. Age and gender were forced in the model.


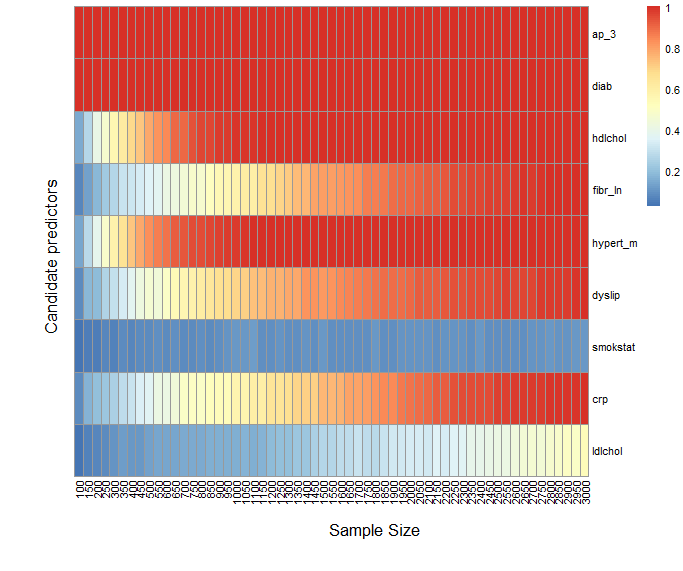


**Figure S8**. Bootstrap-corrected vs Holdout sample predictive performance for the ovarian cancer data (left) and the CAD (right) for the RCS strategy.


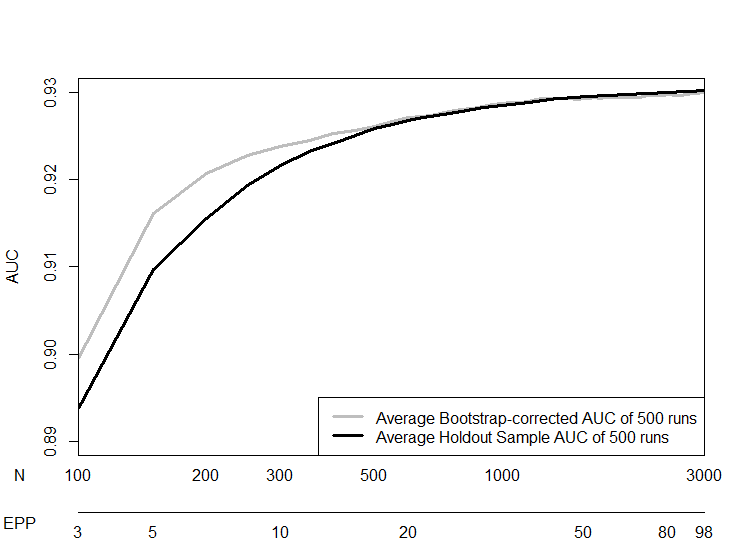

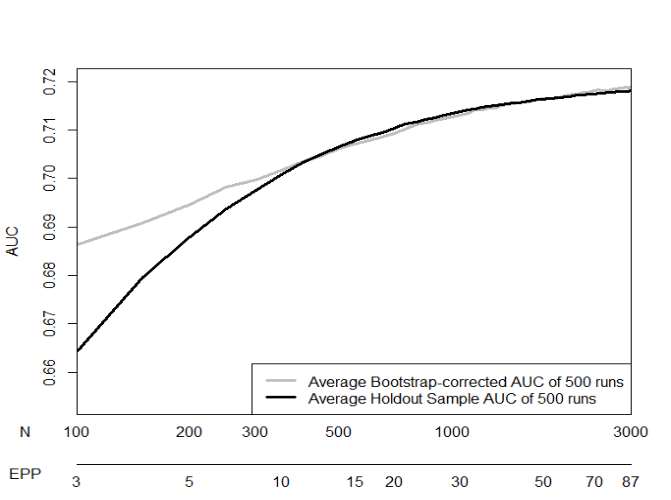


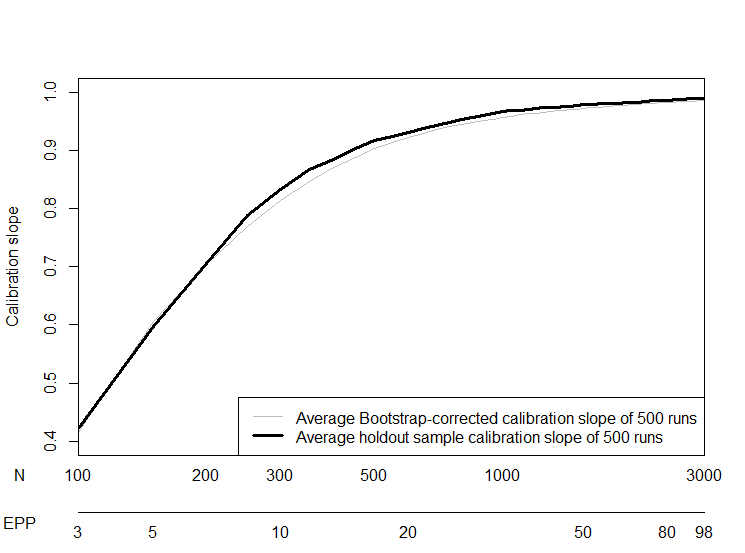

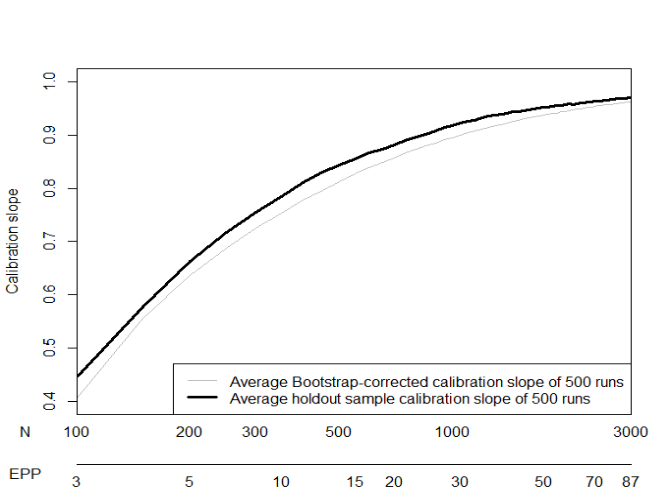


**Figure S9**. Bootstrap-corrected vs Holdout sample predictive performance for the ovarian cancer data (left) and the CAD (right) for the Firth strategy.


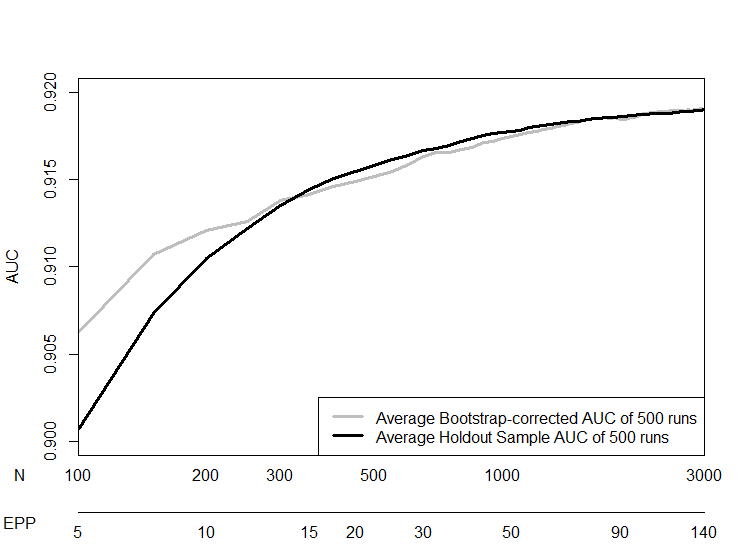

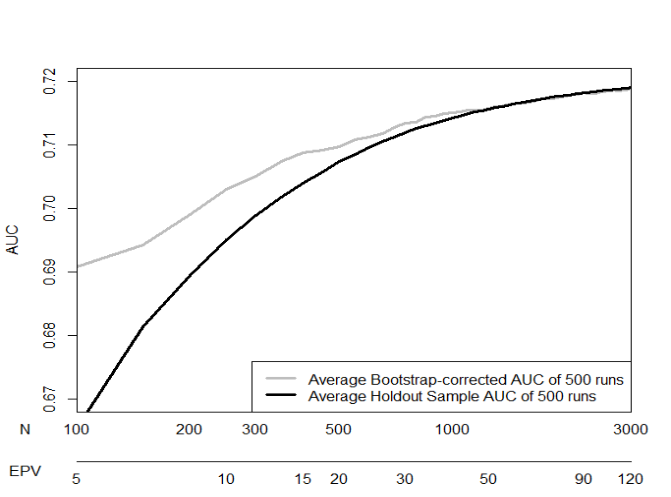


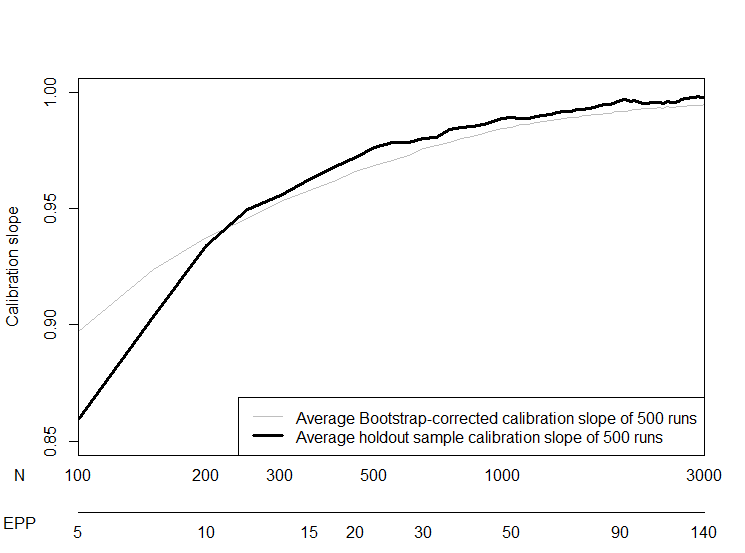

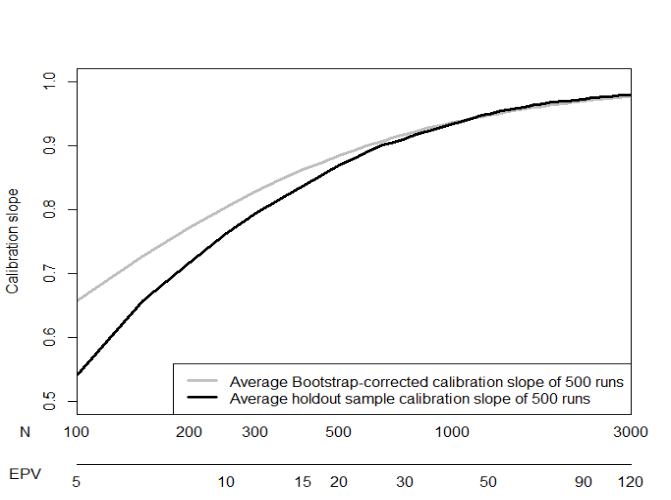


**Figure S10**. Bootstrap-corrected vs Holdout sample predictive performance for the ovarian cancer data (left) and the CAD (right) for the variable selection strategy


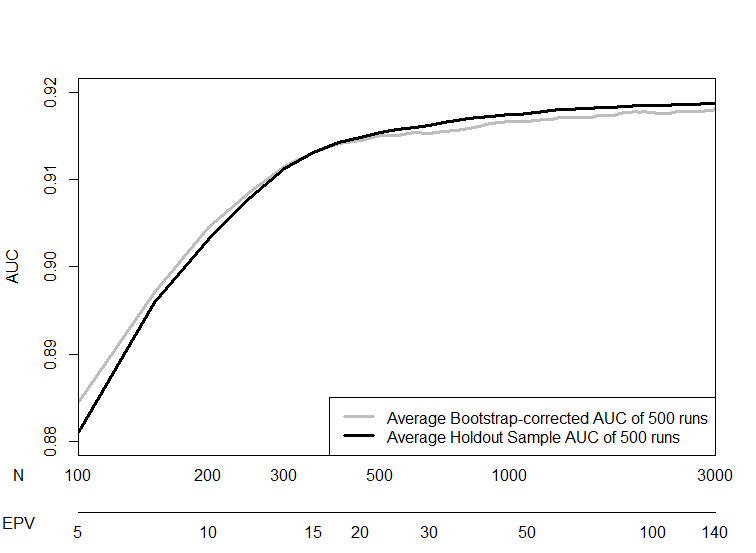

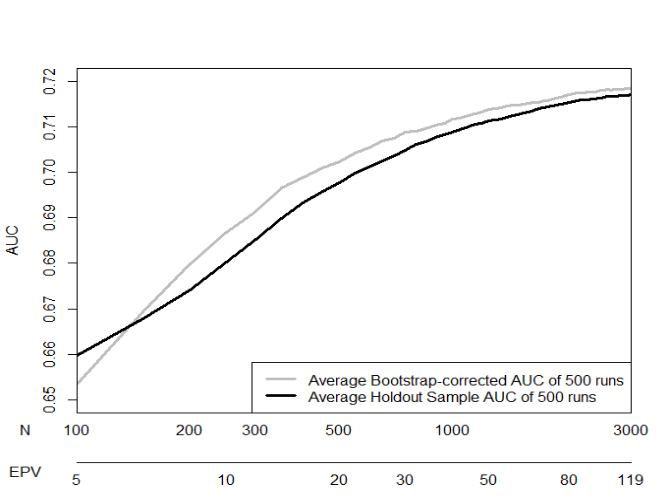

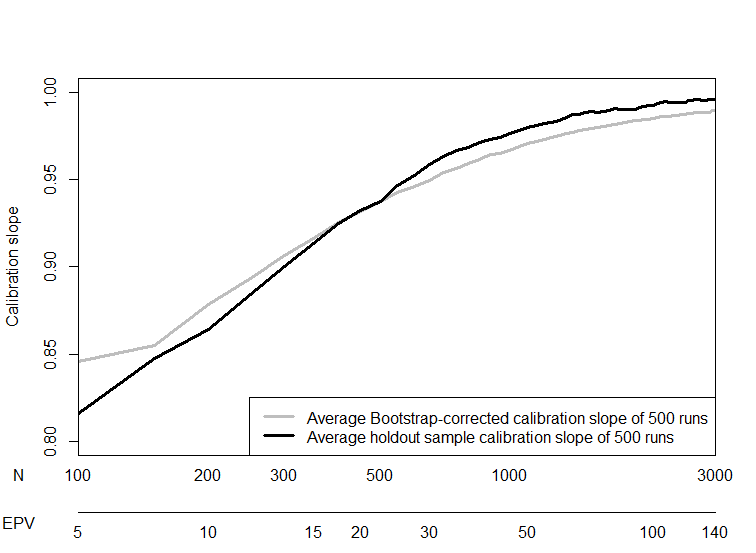

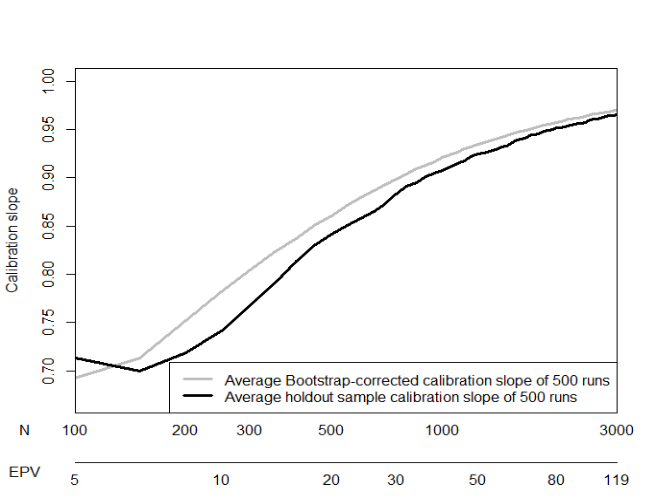


**Figure S11**. Apparent AUC based on sampling with replacement (Panel A), apparent AUC based on sampling without replacement (Panel B), and Standard Deviation of Apparent AUC when sampling was done with and without replacement (Panel C) for the IOTA data for the basic modeling strategy

A B


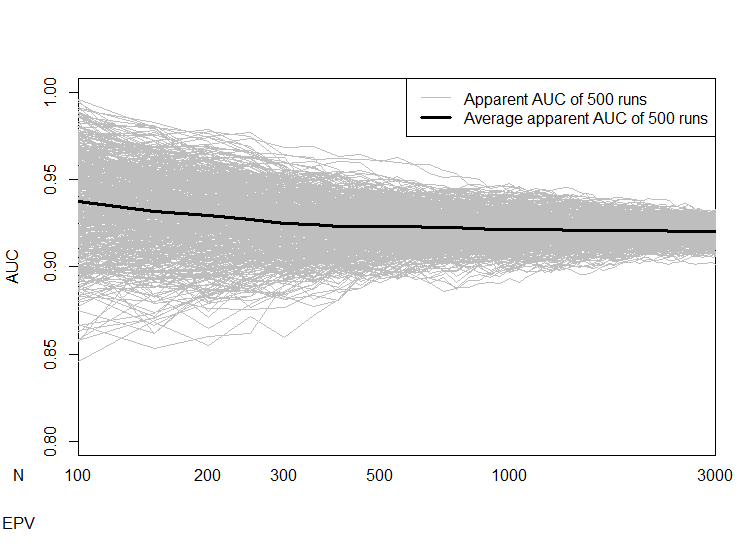

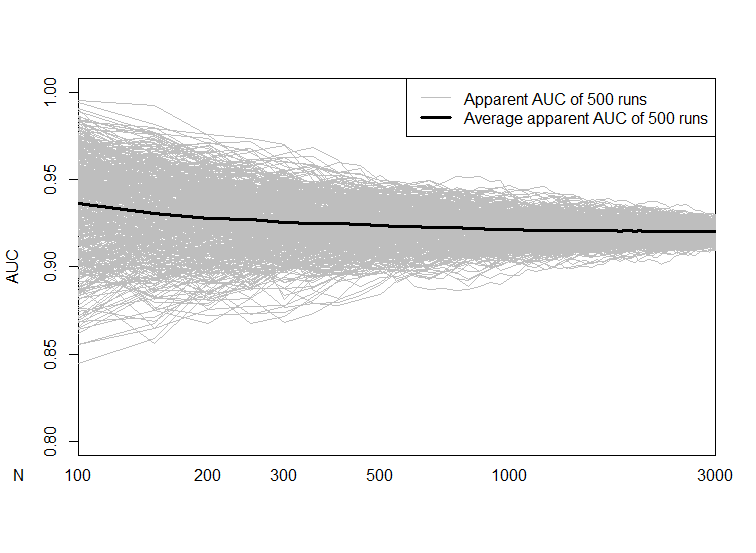


C


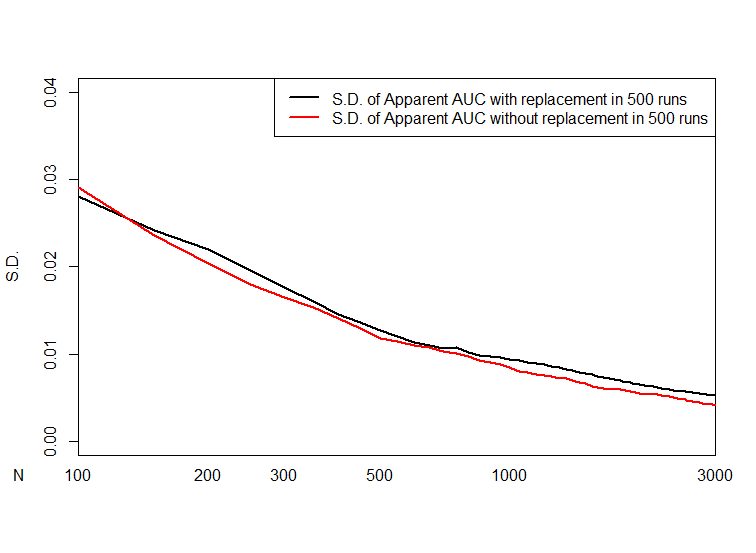


**Figure S12**. Apparent AUC based on sampling with replacement (Panel A), apparent AUC based on sampling without replacement (Panel B), and Standard Deviation of Apparent AUC when sampling was done with and without replacement (Panel C) for the CAD data for the basic modeling strategy

A B


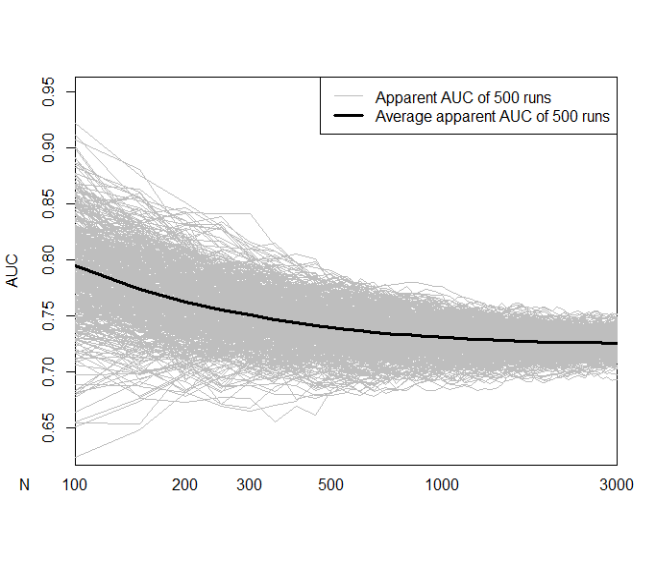
**
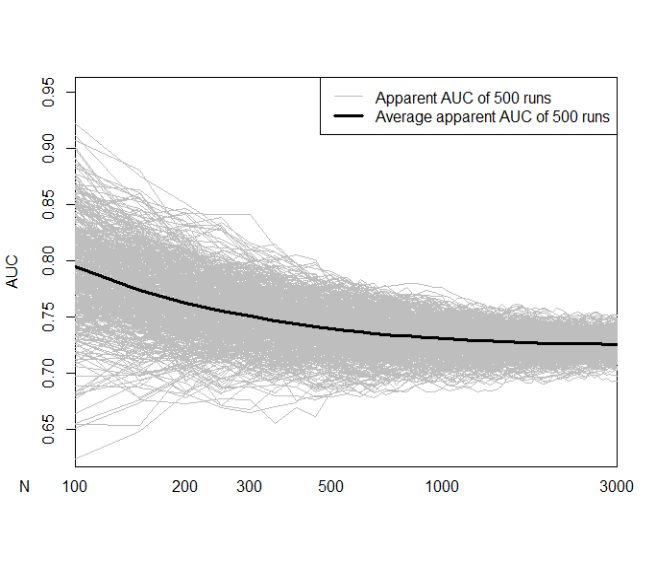
**

C

**
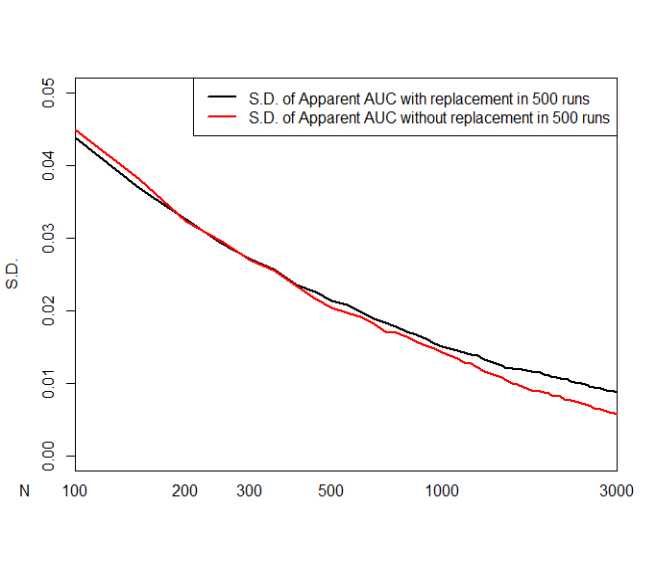
**

## **References**

1. Wahl S, Boulesteix A-L, Zierer A, Thorand B, van de Wiel MA. Assessment of predictive performance in incomplete data by combining internal validation and multiple imputation. BMC Med Res Methodol. 2016;16(1):144.

2. Harrell Jr FE. Regression modeling strategies: with applications to linear models, logistic and ordinal regression, and survival analysis. Springer; 2015.

3. Steyerberg EW. Clinical prediction models. Springer; 2019.

4. Steyerberg EW, Harrell Jr FE, Borsboom GJJM, Eijkemans MJC, Vergouwe Y, Habbema JDF. Internal validation of predictive models: efficiency of some procedures for logistic regression analysis. J Clin Epidemiol. 2001;54(8):774–81.

5. Riley RD, Ensor J, Snell KIE, Harrell FE, Martin GP, Reitsma JB, et al. Calculating the sample size required for developing a clinical prediction model. Bmj. 2020;368.

6. Timmerman D, Bourne TH, Tailor A, Collins WP, Verrelst H, Vandenberghe K, et al. A comparison of methods for preoperative discrimination between malignant and benign adnexal masses: the development of a new logistic regression model. Am J Obstet Gynecol. 1999;181(1):57–65.

7. Su JQ, Liu JS. Linear Combinations of Multiple Diagnostic Markers. J Am Stat Assoc. 1993;88(424):1350–5.

8. Genders TSS, Steyerberg EW, Hunink MGM, Nieman K, Galema TW, Mollet NR, et al. Prediction model to estimate presence of coronary artery disease: retrospective pooled analysis of existing cohorts. Bmj. 2012;344:e3485.
